# DNA sequence and chromatin differentiate sequence-specific transcription factor binding in the human malaria parasite *Plasmodium falciparum*

**DOI:** 10.1093/nar/gkae585

**Published:** 2024-07-05

**Authors:** Victoria A Bonnell, Yuning Zhang, Alan S Brown, John Horton, Gabrielle A Josling, Tsu-Pei Chiu, Remo Rohs, Shaun Mahony, Raluca Gordân, Manuel Llinás

**Affiliations:** Department of Biochemistry and Molecular Biology, The Pennsylvania State University, University Park, PA 16802, USA; Huck Institutes Center for Eukaryotic Gene Regulation, The Pennsylvania State University, University Park, PA 16802, USA; Huck Institutes Center for Malaria Research, The Pennsylvania State University, University Park, PA 16802, USA; Center for Genomic and Computational Biology, Duke University, Durham, NC 27708, USA; Department of Biostatistics and Bioinformatics, Duke University, Durham, NC 27708, USA; Program in Computational Biology and Bioinformatics, Duke University, Durham, NC 27708, USA; Department of Biochemistry and Molecular Biology, The Pennsylvania State University, University Park, PA 16802, USA; Huck Institutes Center for Eukaryotic Gene Regulation, The Pennsylvania State University, University Park, PA 16802, USA; Huck Institutes Center for Malaria Research, The Pennsylvania State University, University Park, PA 16802, USA; Center for Genomic and Computational Biology, Duke University, Durham, NC 27708, USA; Department of Biostatistics and Bioinformatics, Duke University, Durham, NC 27708, USA; Department of Biochemistry and Molecular Biology, The Pennsylvania State University, University Park, PA 16802, USA; Huck Institutes Center for Eukaryotic Gene Regulation, The Pennsylvania State University, University Park, PA 16802, USA; Huck Institutes Center for Malaria Research, The Pennsylvania State University, University Park, PA 16802, USA; Department of Quantitative and Computational Biology, University of Southern California, Los Angeles, CA 90089, USA; Department of Quantitative and Computational Biology, University of Southern California, Los Angeles, CA 90089, USA; Department of Chemistry, University of Southern California, Los Angeles, CA 90089, USA; Department of Physics and Astronomy, University of Southern California, Los Angeles, CA 90089, USA; Thomas Lord Department of Computer Science, University of Southern California, Los Angeles, CA 90089, USA; Department of Biochemistry and Molecular Biology, The Pennsylvania State University, University Park, PA 16802, USA; Huck Institutes Center for Eukaryotic Gene Regulation, The Pennsylvania State University, University Park, PA 16802, USA; Center for Genomic and Computational Biology, Duke University, Durham, NC 27708, USA; Department of Biostatistics and Bioinformatics, Duke University, Durham, NC 27708, USA; Department of Computer Science, Duke University, Durham, NC 27708, USA; Department of Molecular Genetics and Microbiology, Duke University, Durham, NC 27708, USA; Department of Biochemistry and Molecular Biology, The Pennsylvania State University, University Park, PA 16802, USA; Huck Institutes Center for Eukaryotic Gene Regulation, The Pennsylvania State University, University Park, PA 16802, USA; Huck Institutes Center for Malaria Research, The Pennsylvania State University, University Park, PA 16802, USA; Department of Chemistry, The Pennsylvania State University, University Park, PA 16802, USA

## Abstract

Development of the malaria parasite, *Plasmodium falciparum*, is regulated by a limited number of sequence-specific transcription factors (TFs). However, the mechanisms by which these TFs recognize genome-wide binding sites is largely unknown. To address TF specificity, we investigated the binding of two TF subsets that either bind CACACA or GTGCAC DNA sequence motifs and further characterized two additional ApiAP2 TFs, PfAP2-G and PfAP2-EXP, which bind unique DNA motifs (GTAC and TGCATGCA). We also interrogated the impact of DNA sequence and chromatin context on *P. falciparum* TF binding by integrating high-throughput *in vitro* and *in vivo* binding assays, DNA shape predictions, epigenetic post-translational modifications, and chromatin accessibility. We found that DNA sequence context minimally impacts binding site selection for paralogous CACACA-binding TFs, while chromatin accessibility, epigenetic patterns, co-factor recruitment, and dimerization correlate with differential binding. In contrast, GTGCAC-binding TFs prefer different DNA sequence context in addition to chromatin dynamics. Finally, we determined that TFs that preferentially bind divergent DNA motifs may bind overlapping genomic regions due to low-affinity binding to other sequence motifs. Our results demonstrate that TF binding site selection relies on a combination of DNA sequence and chromatin features, thereby contributing to the complexity of *P. falciparum* gene regulatory mechanisms.

## Graphical Abstract

**Graphical Abstract:**
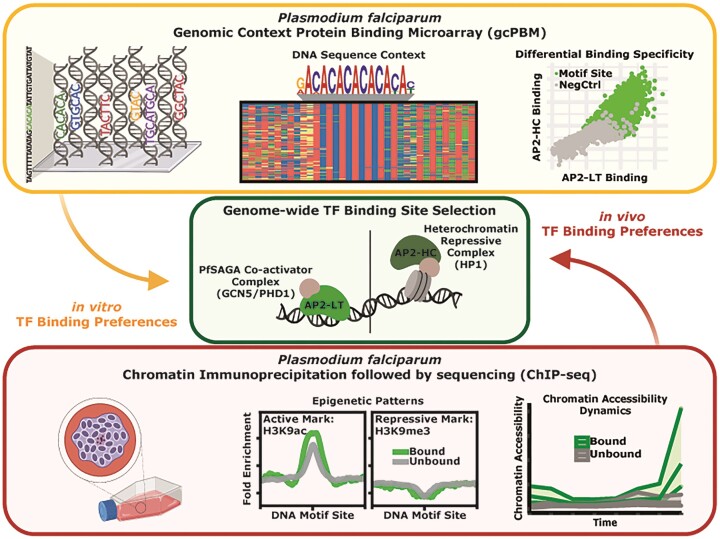

## Introduction

Sequence-specific transcription factors (TFs) bind a core DNA sequence motif through base-specific contacts using the major and/or minor groove of DNA (1–6). Eukaryotic TFs often form base-specific contacts with short DNA motifs (6-8mers), with additional preferences to adjacent sequence context through non-base-specific interactions (1–8). However, the nature of these protein-DNA interactions is more complex than simply recognizing a specific DNA motif, since, for any given TF, only a fraction of the total possible genome-wide sites are bound (1,7–9). Numerous features such as DNA sequence context, local DNA topography, post-translational modifications (PTMs) of the TF or histones, TF protein concentration, TF timing of expression, DNA methylation patterns, protein-interaction partners, and chromatin state can all influence TF binding site recognition (1,5,10–17). Since dysregulation of TF binding can be deleterious such as in cancer (i.e. p53 and MYC) (18,19) or can impact stress tolerance in plant crops (*i.e*. AP2/ERFs) (20–23), determining how individual TFs select and bind to cognate DNA motifs *in vivo* is central to understanding gene regulatory networks.

In general, eukaryotes encode expanded TF families that arise through gene duplication events and subsequent diversification, resulting in evolutionarily conserved DNA-binding domains (DBDs) that bind highly similar DNA sequences (i.e. paralogous domains) (1,7,8,24,25). Paralogous TFs are highly represented in model eukaryotes from unicellular (e.g. yeast) to multicellular (e.g. mammals) organisms, have been shown to function in both unique or redundant manners, and govern alternate transcriptional regulatory networks in different cell types (25–27). The most well-studied example is the homeobox domain (HOX) TF family in animals, which contains many paralogous TFs that all recognize A/T-rich DNA motifs (28). While HOX TFs recognize similar DNA motifs *in vitro*, they have highly divergent *in vivo* functions. This specificity is largely due to moderate- and low-affinity binding events driven by a combination of factors including the DNA sequence context surrounding the A/T-rich HOX motifs, interactions with divergent co-factors inducing latent specificities, and varying abilities to bind inaccessible chromatin (11,17,28–30).

Single-celled eukaryotes, such as yeast, have served as models to explore the unique expansion of paralogous TF binding in the absence of multicellularity (31–36). Yeast paralogous TFs generally regulate vastly differing target genes, often in response to diverse extracellular environments (37,38). Similarly, single-celled, eukaryotic Apicomplexan parasites encode TFs with paralogous DBDs. However, the number of paralogous domains in apicomplexans are drastically reduced due to genome reduction following evolutionary adaptation to a parasitic lifestyle (39,40). The Apicomplexan human malaria parasite, *Plasmodium falciparum*, presents a unique opportunity to explore the challenge of paralogous protein–DNA specificity as it has evolved surprisingly few TFs and possesses a limited repertoire of sequence-specific TFs (41,42). These TFs are thought to act through an array of unique DNA sequence motifs found in regulatory regions upstream of transcription start sites (TSSs) (41–47) in the context of a 22.9 Mb genome that is one of the most A/T-rich genomes sequenced to date (ranging from 85% genome-wide up to 90% A/T in intergenic regions) (43).

The *P. falciparum* genome encodes thirteen homeodomain-like (HD) (48) proteins, ten myeloblastosis (MYB) (49,50) proteins, three high mobility group box (HMGB) (51,52) domain-containing proteins as well as an expanded set of 170 diverse zinc finger domain proteins (53–55) that are largely uncharacterized to date (43,44,46). The largest and best characterized family of *P. falciparum* TFs (<30 members) is the Apicomplexan APETALA2 (ApiAP2) family of DNA-binding proteins that contain one to three AP2 DBDs (41,42,56–58). APETALA2/Ethylene response factor (AP2/ERF) TFs are also one of the most highly represented TF families in plant-lineage genomes (58) and have no mammalian counterparts. AP2/ERF protein-DNA recognition occurs via three anti-parallel beta-strands directly interacting with the DNA major groove, a recognition modality that is consistent in both plant and Apicomplexan AP2/ERF protein–DNA complexes (61–67). Plant AP2/ERFs have highly paralogous DBDs that all recognize a GCC-box DNA motif, with well-defined activation and repression domains (23,61–63). In contrast, ApiAP2 proteins have more divergent AP2 domains that recognize a wide variety of over 20 unique DNA motifs and contain surprisingly few additional functional domains (41,56,57).

*P. falciparum* critically relies on precise regulation of gene expression throughout its lifecycle (59,60,68–70). In addition to the 48-hour asexual replicative cycle in human erythrocytes, *P. falciparum* parasites undergo several major developmental stages. These include sexual development for transmission between human and mosquito, growth and replication in the mosquito midgut, maturation in the mosquito salivary glands, and replication in human hepatocytes. These transformations all rely on the action of sequence-specific TFs in concert with epigenetic regulation (59,60) to program cellular differentiation, genome maintenance, immune system evasion, and development of the malaria parasite (57,58). In this study, we interrogated the relevance of both DNA sequence and chromatin context on the genomic binding site selection of several paralogous and non-paralogous DBDs from TFs in *P. falciparum*. To do this, we generated a novel *P. falciparum*-specific genomic-context protein-binding microarray (gcPBM). Using the gcPBM, we simultaneously probed protein-DNA interactions between DBDs and all intergenic instances of its cognate DNA motif directly from the *P. falciparum* genome (10,12). We also used comparative bioinformatic analyses to explore features of the chromatin environment such as genome-wide TF occupancy, chromatin accessibility, and epigenetic histone PTMs (47,71–73). Our findings suggest that a subset of *P. falciparum* TFs are greatly impacted by DNA sequence context while others are influenced by a complex interplay between TF timing of expression, genome-wide occupancy, and the chromatin landscape. Understanding ApiAP2 DNA-binding selectivity has direct implications for targeting the regulation of parasite development as a potential for new therapeutic intervention strategies.

## Materials and methods

### Protein induction and affinity purification

**Figure 1.**
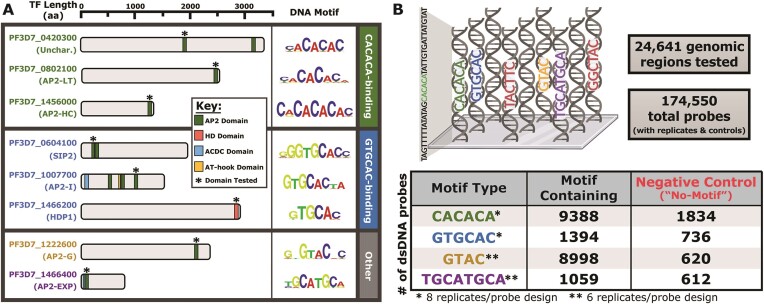
Multiple *P. falciparum* TFs with overlapping sequence preferences and design of the *P. falciparum* genomic-context protein-binding microarray (gcPBM). (**A**) Graphical representation of each TF examined in this study categorized into ‘CACACA-binding’, ‘GTGCAC-binding’, and ‘Other’. Protein lengths (in number of amino acids) are drawn to scale. Predicted protein domains were determined using NCBI Conserved Domain Search or defined by previous literature. Position weight matrix (PWM) logos are from previously published universal protein-binding microarray (PBM) experiments. * Denotes the DNA-binding domains (DBDs) tested in this study; (**B**) Graphical representation of the *P. falciparum* gcPBM design. Position weight matrix (PWM) data was searched against intergenic regions of the *P. falciparum* genome (Pfalciparum3D7; version 3, release 38) and categorized into four motif types (CACACA, GTGCAC, GTAC and TGCATGCA). All sequences were replicated eight(*) or six(**) times. Microarray graphic was created with BioRender.com.

All experiments were conducted using purified AP2 DNA-binding domains (DBDs) (starred [*] in Figure 1A; Supplementary Figure S1A) fused to an N-terminal glutathione-S-transferase (GST) tag and was purified as demonstrated previously (41,42). Briefly, each AP2 domain was previously cloned into the pGEX-4-T1 vector (GE Life Sciences) and transformed into BL21-CodonPlus (DE3)-RIL *E. coli (*Stratagene) for protein production using Isopropyl β-d-1-thiogalactopyranoside (IPTG) induction (41). Verification of a successful GST-tagged AP2 protein purification by GST-affinity purification (Thermo Scientific Pierce Glutathione Superflow Agarose beads) was demonstrated by running protein samples on 4-15% stacking SDS-PAGE gel and stained with Coomassie Blue. Integrity of the GST tag was subsequently checked via western blotting of the purified recombinant protein using anti-GST antibodies (Invitrogen 71-7500) [1:1,500 dilution]. The homeodomain from HDP1 was tagged and purified similarly with modifications (48) and gifted to us for the gcPBM experiments from the Kafsack lab at Cornell University.

### Universal protein-binding microarray (PBM) for quality control

Each purified GST-tagged DBD stock was validated for DNA-binding specificity using 4 × 44k universal protein-binding microarrays (PBMs) (Agilent Technologies), as described previously (10,41,42,48,74,75), before testing on the *Plasmodium falciparum*-specific genomic-context protein-binding microarray (gcPBM) design. The experiments were repeated, as previously demonstrated (41,42), with the exception that they were tested on protease-treated, re-used arrays, to determine the amount of protein necessary to generate signal within the linear range of the GenePix 4300B Microarray Scanner (Molecular Devices) at a resolution of 5μm. In brief, the microarray stripping protocol (74) includes: (1) an overnight incubation in a protease solution (350 units of protease [Sigma 537088], 10% v/v SDS, and 10 mM EDTA), three washes in 1x PBS with 0.5% v/v Tween20, a final rinse in 1× PBS, then scanning for Alexa488 signal to ensure digestion of protein from the previous experiment. The PBM method includes 5 major steps (74) with 1xPBS with 0.01-0.5% v/v Tween20 washes in between each step: (1) Blocking the microarray with 2% w/v milk in 1× PBS for one hour in the dark, (2) Applying the protein-binding mixtures to the microarray for one hour, (3) Probing protein-DNA interactions with an anti-GST, rabbit IgG, Alexa488-conjugated antibody (1:40 dilution in 2% w/v milk blocking solution; Invitrogen, A11131) for one hour, (4) Scanning the microarray for Alexa488 signal (GenePix Pro version 7.2 software), and (5) Identifying enriched DNA motif using a gapped 8-mer DNA motif-searching perl script modified from original script (74) to normalize only based on local neighboring probes instead of Cy3 signal introduced from double-stranding the microarray.

### Design of *Plasmodium falciparum* genomic-context protein-binding microarray (gcPBM)

Using position weight matrix (PWM) data from published work (41,42), all instances of each motif were identified in the *P. falciparum* genome (*Plasmodium falciparum* 3D7 strain genome release v38 (76)), using a motif *E*-score cutoff of >0.45 (10,12). Only intergenic regions (excluding telomeric regions) were used for this gcPBM design. The numbers of probes found per DNA motif are as follows: 2848 probes with putative sites for PF3D7_0420300_D1 (500 negative controls), 4251 probes with putative sites for AP2-LT (500 negative controls), 3864 probes with putative sites for PF3D7_1305200 (500 negative controls), 4321 probes with putative sites for AP2-HC (500 negative controls), 1459 probes with putative sites for SIP2_D1 (500 negative controls), 3742 probes with putative sites for AP2-I_D3 (500 negative controls), 8998 probes with putative sites for AP2-G (1000 negative controls), and 1059 probes with putative sites for AP2-EXP (1000 negative controls). Negative control probes were randomly selected, unique, intergenic sites with similar nucleotide content as the motif-containing sites, but did not contain the motif-of-interest (e.g. CACACA negative control probes did not contain the CACACA motif and GTGCAC negative control probes did not contain the GTGCAC motif). HDP1 motif-specific genomic sequences were not initially included in the gcPBM due to its identification and characterization (48) after the initial design of the gcPBM experiment, but was included due to PWM similarities to SIP2_D1 and AP2-I_D3. Any sequence containing another instance of the centered motif in the left or right flanks was mutated to prevent multiple binding sites per 36-bp window. Due to the similarities between the CACACA and GTGCAC PWMs, there were genomic DNA sequences that led to redundant probe designs, which were discarded, leaving only one instance of the sequence. After discarding redundant probe designs with motif types, the total number of probes per motif type was as follows: 9388 probes with putative CACACA sites (1834 CACACA negative controls); 1394 probes with putative GTGCAC sites (736 GTGCAC negative controls); 8998 probes with putative GTAC sites (620 GTAC negative controls); and 1059 probes with putative TGCATGCA sites (612 TGCATGCA negative controls). Overall, the *P. falciparum* gcPBM design reached a total of 24,641 unique genomic regions (Supplemental File 1). Each double stranded DNA probe was represented in both the 5′ and 3′ orientations, with one end of each DNA molecule attached to a glass slide (Supplementary Figure S2A). Additionally, each DNA probe was replicated in random areas of the microarray surface (four CACACA/GTGCAC replicates and three GTAC/TGCATGCA replicates per orientation), which brought the total number of DNA probes to 174,550 spots for a 4 × 180k microarray (Agilent Technologies). Additional spots on the array were set aside for control grid alignment, microarray scanning, and downstream analysis.

### Genomic-context protein-binding microarray (gcPBM) experiment

The single-stranded DNA microarrays were double-stranded by solid-state primer extension as reported previously (10,12). The 24bp primer sequence (5'-GTCTTGATTCGCTTGACGCTGCTG-3') was used to double-strand all gcPBM slides for this study. Each GST-tagged DBD was tested for DNA sequence specificity by applying protein to the *P. falciparum* gcPBM as demonstrated previously (10,12,74). The major components of the protein-binding mixture are as follows: 1% w/v milk, 0.2 mg/ml BSA, 0.5% v/v salmon testes DNA, and 0.03% v/v TritonX-100 in 1× PBS (10,12,13,74,75). Amounts of recombinant protein necessary for DNA-binding was empirically determined during preliminary universal PBM experiments detailed above (average amount added is around 25 μg; identified by *A*_280_ signal on NanoDrop 2000 Spectrophotometer.

### Genomic-context protein-binding microarray (gcPBM) data acquisition and analysis

Immediately after completing the gcPBM experiment, each microarray chamber was scanned using the GenePix 4400A Microarray Scanner (Molecular Devices) at a resolution of 2.5 μm, with the 488 nm wavelength laser, along with the GenePix Pro7 software to generate image files (.tif). The image files were aligned with the GenePix Array List (.gal) file to associate raw signal intensity to the *P. falciparum* genome-derived DNA sequences, which generates a GenePix Results (.gpr) file. The .gpr files were then further processed and normalized using the previously published Masliner script and downstream analysis made by the Bulyk lab and modified by the Gordân lab (10,12,13,74). Final values used for this study are a single data point representing the highest natural log median binding intensity value across the replicates between either the 5′ or 3′ orientations for each DNA sequence.

### Validation with electrophoretic mobility shift assays (EMSAs)

All gel shift experiments were conducted using the LightShift Chemiluminescent EMSA kit and protocol (Thermo Scientific), with some modifications. DNA sequences designed for each gel shift are found in Supplemental File 2. Single-stranded biotinylated DNA oligos were annealed to their reverse complement sequence to generate double-stranded DNA probes using the Duplex Buffer recipe (100 mM Potassium acetate, 30 mM HEPES [pH 7.5]) and protocol from Integrated DNA Technologies (IDT) website. The minimal concentration of protein added to the binding reaction to produce a robust shift was empirically determined by titrating in varying amounts of protein with the wildtype DNA sequences (data not shown). The standard protein-binding mixture includes: 1× binding buffer (100 mM Tris–HCl [pH 7.5], 500mM KCl, 10mM DTT), 5mM MgCl_2_, 25ng/μl Poly dI-dC, 0.05% v/v NP-40, protein in 25% v/v glycerol, and biotinylated dsDNA probe. Briefly, the protocol includes: (i) Combining all components of the protein-binding mixture and incubating at room temperature for 20 min, (ii) running samples on a pre-run 6% non-denaturing PAGE gel using 0.5× TBE, (iii) transfer DNA onto a nylon membrane using 0.5× TBE, (iv) crosslink the nylon membrane at 312 nm for 15 min with a transilluminator, (v) Block the membrane for 15 min with shaking, (vi) Detect the biotin-labeled DNA by added Stabilized Streptavidin-Horseradish Peroxidase Conjugate (1:300 dilution) for 15 min with shaking in blocking buffer, (vii) wash the membrane 4× with shaking in 1× wash buffer, (viii) equilibrate the membrane with shaking and (ix) incubate with the substrate working solution (1:1 luminol enhancer/peroxide) without shaking. Final images taken on ChemiDoc XRS + Molecular Imager (Bio-Rad). Exposure times determined by minimal exposure without oversaturation.

### Designing DNA oligos with predicted DNA shape mutations

The DNA oligos used to investigate the impact of predicted *in vitro* DNA shape on binding were generated by the mutation design tool of TFBSshape (77). TFBSshape produces oligo sequences with mutations that minimize DNA sequence changes while generating dynamics changes to the predicted shape features according to the distance between their wild type and mutant. The sequence distance is determined by Levenshtein distance that sums the number of substitutions, deletions or insertions required to transform from a mutant to its wild type sequence. The predicted shape distance is calculated in Euclidean distance between two normalized shape feature vectors for a wild type and its mutant sequence. The normalized predicted shape features including helix twist (HelT), minor groove width (MGW), propeller twist (ProT), and roll are derived from DNAshapeR (78). Three bp on the flanks of the fixed core AGTGCATTA were subjected to mutation, as shown in lowercase in Supplemental File 3. The oligos with the maximum shape distance, with respect to the preserved sequence distance were selected. The sequence distance was preserved, and the predicted shape distance was calculated and sorted among all possible mutations.

### Generation of *Plasmodium falciparum* parasite line and culturing conditions

The endogenous locus of *pfap2-lt* was modified using CRISPR-Cas9 to insert a sequence encoding a C-terminal (3x) hemagglutinin (HA) tag (Supplementary Figure S7) to generate an AP2-LT^HA^ tagged parasite line. Using a 700 bp homology region with a 3× HA sequence left-flanked by the 3′-end of AP2-LT coding sequence and right-flanked by the endogenous 3′-untranslated region (UTR) of AP2-LT was cloned into the pDC2-U6A-hDHFR vector. This CRISPR single-plasmid design contained a *pfap2-lt* locus-targeted guide RNA sequence with BbsI restriction site, Cas9^HA^, AMP^R^, WR^R^ and NotI and SacI restriction sites flanking the homology region. The final plasmid was transformed into heat shock competent DH5α cells with 100μg/ml ampicillin (AMP). Then the plasmid was purified and ethanol precipitated overnight at -80°C. Wild type parasites (3D7 strain) were cultured under standard *P. falciparum* culturing conditions (5% O_2_, 7% CO_2_, 37°C, RPMI 1640 media with 0.5% AlbumaxII and hypoxanthine). The purified plasmid DNA (100 μg) was transfected by electroporating uninfected erythrocytes and adding trophozoite-stage parasites to later invade the erythrocytes preloaded with plasmid DNA. After parasite reinvasion, parasites were treated with 2.5 nM of WR99210 for one week and switched to no drug standard parasite media. After limited dilution cloning to generate a clonal parasite line, genomic DNA was purified from the AP2-LT^HA^ parasite culture (Qiagen) and verified for integration by PCR (primer sequences in Supplemental File 2; Supplementary Figure S7), Sanger sequencing, and whole genome sequencing via high-throughput Illumina sequencing.

### AP2-LT chromatin immunoprecipitation followed by high-throughput sequencing (ChIP-seq)

Three biological replicates of ChIP-seq using a clonal parasite line of AP2-LT^HA^ and a negative control no-epitope sample were conducted at the peak of AP2-LT protein expression during the early schizont stage (36–45 h post invasion [hpi]) (Supplementary Figure S8). The ChIP-seq experiment was carried out using a similar protocol as previous published work (79). The ChIP-seq protocol had five major steps including: (i) Chemically crosslinking the all protein-protein and protein-chromatin interactions, (ii) Isolation of parasite nuclei, (iii) Parasite nuclei lysis with chromatin sonication, (iv) protein–chromatin complex immunoprecipitation and (v) DNA purification. The crosslinking step included: (a) chemical crosslinking suspended *Pf*AP2-LT^HA^ (or WT culture for negative control experiment) parasite culture (at least 10^8^ 36–45 hpi schizont-stage parasites synchronized with 10% w/v sorbitol more than one cycle prior) with 1% v/v formaldehyde for 10 min at 37°C, (b) quench the crosslinking with 125 mM glycine on ice for 5 min, (c) pellet the cells by centrifugation and lyse the red blood cells with 0.1% w/v saponin in 1× PBS and (d) wash the cells by repeated centrifugation with 1xPBS to remove the red blood cell debris. The nuclei isolation step included: (a) resuspend parasite pellet with 10^9^ schizonts/2 ml lysis buffer (10 mM HEPES [pH 7.9], 10 mM KCl, 0.1 mM EDTA [pH 8.0], 0.1 mM EGTA [pH 8.0], 1 mM DTT (added just before using) and 1× protease inhibitors) and incubate on ice for 30 min, (b) add a final concentration of 0.25% v/v NP-40 and incubate for 1 min, (c) Lyse parasite membranes with pre-chilled glass dounce homogenizer for 100 strokes per 10^9^ schizonts/2 ml Lysis Buffer and (d) pellet the nuclei and freeze pellet at –80°C overnight. The sonication step included: (a) Resuspend parasite nuclei pellet in 500 μl of Shearing Buffer (0.1% v/v SDS, 10mM Tris [pH 8.0], 1mM EDTA, and 1x protease inhibitors) per 5 × 10^8^ parasites and (b) sonicate the chromatin until sufficiently sheared (130 μl, 5% duty cycle, 75 W peak incident power, 200 cycles per burst, 7°C, for 5 min using Covaris Focus-Ultrasonicator M220). The immunoprecipitation step included: (a) dilute the sample 1:5 with dilution buffer (0.01% v/v SDS, 1.1% Triton X-100, 1.2 mM EDTA, 16.7 mM Tris–HCl [pH 8.1] and 150 mM NaCl), (b) reduce background signal by pre-clearing with 20 μl of protein A/G magnetic beads (Millipore 16-663) per 1 ml of sample for 2 h at 4°C with rotation, (c) Aliquot 1/10 of the sample for the Input control and rotate at 4°C until DNA elution step, (d) Remaining 9/10 of sample is immunoprecipitated with 1:1000 anti-HA antibody (0.1mg/ml Roche Rat Anti-HA High Affinity [11867423001]) overnight at 4°C with rotation, (e) Collect the immune complexes with 20μl of Protein A/G magnetic beads per 1ml of sample for 2 hours at 4°C with rotation, (f) Wash (5 min each) immune complexes on magnet once with Low Salt Immune Complex Wash Buffer (0.1% v/v SDS, 1% v/v TritonX-100, 2 mM EDTA, 20 mM Tris–HCl [pH 8.1], and 150mM NaCl) at 4°C with rotation, once with High Salt Immune Complex Wash Buffer (0.1% v/v SDS, 1% v/v Triton X-100, 2 mM EDTA, 20 mM Tris–HCl [pH 8.1] and 500 mM NaCl) at 4°C with rotation, once with LiCl Immune Complex Wash Buffer (0.25 M LiCl, 1% v/v NP-40, 1%w/v deoxycholate, 1 mM EDTA and 10 mM Tris–HCl [pH 8.1]) at 4°C with rotation, and twice with 1× TE at room temperature with rotation, and (g) Elution from beads with 200 μl fresh Elution Buffer (0.01% v/v SDS and 100 mM NaHCO_3_) per 1ml of sample for 15 min at room temperature with rotation (also added to Input sample). The DNA purification step included: (a) reverse-crosslink Input and IP samples with 0.2 M NaCl and incubate overnight at 45°C with shaking (750 rpm), (b) 0.4% v/v RNaseA treat from 30 min at 37°C with shaking (750 rpm), (c) 0.3 mg/ml Proteinase K treat for 2 h at 45°C with shaking (750 rpm) and (d) purify DNA using MinElute column (Qiagen) as directed by manufacturer. Purified DNA samples were quantified using the Qubit fluorometer for high-sensitivity DNA.

### AP2-LT ChIP-seq library prep for Illumina sequencing

DNA sequencing libraries were prepared for high-throughput Illumina sequencing on the NextSeq 2000 with 150 × 150 single-end or paired-end mode. The library prep protocol includes six major steps: (i) end repair of DNA fragments, (ii) addition of A-tail for adaptor ligation, (iii) ligation of Illumina adaptors, (iv) DNA sequence size selection, (v) DNA sequence amplification by PCR, and (vi) DNA sequence clean up. The single indexed adaptors (Bioo Scientific) used were diluted 1:10 prior to ligation. DNA libraries were size selected for 250 bp sequences. Due to the A/T-richness of the *P. falciparum* genome, KAPA HiFi polymerase was used for amplification during PCR steps. Only 12 rounds of amplification was used for AP2-LT ChIP-seq samples (both for input and IP libraries), and 16 rounds for the no-epitope negative control (both for input and IP libraries). After DNA sequence clean up, completed libraries were quantified using the Qubit fluorometer for high-sensitivity DNA and library sequence length by the Agilent Bioanalyzer 1000 or Agilent TapeStation 4150 before submitting for high-throughput sequencing.

### AP2-LT ChIP-seq data analysis and peak calling

Raw sequencing reads were first processed by trimming (Trimmomatic v0.32.3) Illumina adaptors and low quality reads below 30 Phred (SLIDINGWINDOW: 4:30). FastQC (v0.11.9) was used to check the quality after trimming. Processed reads were then mapped to the to the *P. falciparum* genome (release 38 (76)) using BWA-MEM (v0.7.17.2) simple Illumina mode with multiple mapped reads filtered out (MAPQ = 1). Once the sequences were mapped, MACS2 (80) was used to call peaks with each biological replicate and its paired input sample using a standard significance cutoff (*q*-value = 0.01) (Supplemental File 4). Using BedTools Multiple Intersect (v2.29.2), the narrow peaks output file for each biological replicate was overlapped to identify the significant peaks in at least two of the three replicates (Supplementary Figure S8C). The overlapping regions were then used to identify an enriched DNA sequence motif using Multiple Expectation maximizations for Motif Elicitation (MEME) version 5.5.1 (81) with a minimum motif length of 6 and a maximum length of 25. Target genes were defined by having a peak no more than 2 kb upstream of the gene transcription start site (TSS) (82), within gene bodies, and the closest gene between head-to-head genes. To identify sequence motifs differentially enriched between ChIP-seq peaks and background regions we again used MEME v5.5.1 with the same length parameters (Supplementary Figure S9). Two distinct subsets were analysed: one encompassing all ChIP-seq peaks and another consisting of ChIP-seq peaks that did not overlap with a gcPBM-derived site.

### Integrating gcPBM sequences with ChIP-seq analysis

The gcPBM sequences were mapped as detailed above with the ChIP-seq analysis. BedTools Multiple Intersect (v2.29.2) was used to identify motif-containing gcPBM sequences that were bound via ChIP-seq (‘ChIP-bound’) by setting the minimum overlap at 58.3% of the 36-bp gcPBM sequences, to ensure the overlap contained the central motif (CACACA, GTGCAC, GTAC, TGCATGCA). Of note, the ChIP-unbound sites contained the motif-of-interest centered on the sequence, were from intergenic regions, and contained similar nucleotide composition to the ChIP-bound. The ChIP-unbound sites far outnumbered the ChIP-bound sites by at least 10-fold (Supplementary Figure S11).

### Chromatin accessibility data analysis and comparison to gcPBM and ChIP-seq datasets

All sequences bound below the non-specific binding threshold were excluded and all gcPBM-bound sequences were further characterized into ChIP-bound versus ChIP-unbound by overlapping the mapped gcPBM-bound sequences with ChIP-seq called peaks by MACS2. Overlap was determined using 400 bp windows around the ChIP-seq peak midpoint and the gcPBM midpoint by using bedtools slop and bedtools intersect (bedtools version 2.30.0). Assay for Transposase-Accessible Chromatin using sequencing (ATAC-seq) data (47) was used to assess chromatin accessibility. FASTQ files were downloaded with fastq-dump (version 2.9.6) from GEO (GSE104075) and then aligned to the *P. falciparum* 3D7 reference genome with bwa mem with -M (version 0.7.17). Aligned reads were filtered by mapping quality for 30 with samtools view (version 1.3.1). Then duplicates were removed with picard with commands MarkDuplicates -REMOVE_DUPLICATES true -VALIDATION_STRINGENCY STRICT (version 2.24.1). Each of the timepoints from Toenhake *et al.* 2018 had two ATAC-seq replicates. Additionally, Toenhake, *et al.* provided a control experiment (two replicates) consisting of Tn5-treated genomic DNA (gDNA). These controls were to account for the sequence bias of Tn5. The replicates were merged and then bedgraphs were created from the merged bams with bedtools genomecov with -bg flags and piped into sort -k1,1 -k2,2n. These were smoothed with bedops –chop 100 –stagger 100 and keeping the mean signal in the 100 bp window with bedmap –mean (bedops and bedmap version 2.4.39). Each bin was normalized by dividing the bin's value (representing the average signal in a 100 bp window) by the number of mapped reads in the merged bam file. At the ChIP-seq peak midpoints, the ATAC-seq 100 bp average was divided by the gDNA 100 bp average signal after adding a 0.1 pseudocount to each bin to get the ratio of signal to control.

## Results

### High throughput interrogation of DNA-binding sites for proteins that recognize similar DNA motifs or have overlapping genome-wide occupancies by gcPBM

To explore a possible overlap in binding specificity between *P. falciparum* transcription factors (TFs) we selected three sets of TFs that recognize highly similar DNA sequence motifs. The first set consists of three highly conserved, paralogous AP2 domains that all recognize a CACACA DNA motif (41): PF3D7_0420300, PF3D7_0802100 (PfAP2-LT) (83,84) and PF3D7_1456000 (PfAP2-HC) (85,86) (Figure 1A; Supplementary Figure S1A, B). Next, we selected three non-paralogous *P. falciparum* TFs that all bind a GTGCAC DNA motif including two ApiAP2 proteins, PF3D7_0604100 (PfSIP2) (88,89) and PF3D7_1007700 (PfAP2-I) (79,90), as well as a homeodomain-like protein 1 (PF3D7_1466200; PfHDP1) (48) (Figure 1A; Supplementary Figure S1A, C). Finally, we investigated PF3D7_1222600 (PfAP2-G) (79,91) and PF3D7_1466400 (PfAP2-EXP) (92,93) (Figure 1A), since these TFs have been shown to bind overlapping *in vivo* binding sites (79,92) although they recognize divergent DNA motifs *in vitro* (41,42). While overlapping genomic binding sites have been reported for a number of TFs in *P. falciparum* (79,86,92), the mechanisms that drive these shared binding events are not well understood. These comparative scenarios allowed us to explore the relative contribution of DNA sequence specificity and context to TF binding in *P. falciparum* parasites.

To determine the full spectrum of *in vitro* binding specificities across the *P. falciparum* genome for the eight candidate DBDs, we designed and synthesized a novel *P. falciparum*-specific genomic-context protein-binding microarray (gcPBM) (Figure 1B). This gcPBM design allowed for simultaneous examination of all possible genome-wide motif occurrences for each DBD flanked by *P. falciparum* genomic sequence context. Using position weight matrix (PWM) data from previously published work (41,42), we identified all instances of the DNA motifs, centered on a 36-bp window across all intergenic regions of the *P. falciparum* genome (PlasmoDB 3D7 strain genome release v38 (76); motif E-score cutoff of > 0.45). To assess the non-specific binding of each DBD to the A/T-rich *P. falciparum* genome for each motif type (CACACA, GTGCAC, GTAC, and TGCATGCA), we included negative control DNA probes which contained randomly selected, unique, intergenic genomic sites lacking the motif-of-interest (Figure 1B). After discarding redundant sequences, this resulted in 9388 CACACA probes (1834 negative controls), 1394 GTGCAC probes (736 negative controls), 8998 GTAC probes (620 negative controls), and 1059 TGCATGCA probes (612 negative controls), for a total of 24,641 unique 36-bp genomic sequences (Figure 1B; Supplemental File 1). Each unique DNA sequence was represented in both a 5′ or 3′ orientation on the gcPBM (Supplementary Figure S2A) and was randomly replicated across the gcPBM slide (four CACACA/GTGCAC replicates and three GTAC/TGCATGCA replicates per orientation). Replicate probes were used to calculate the median signal intensity from both probe orientations. Overall, the total number of dsDNA probes was 174 550 spots, which were arrayed using an Agilent Technologies 4 × 180k microarray design.

### CACACA-binding transcription factors show slight preferences for DNA sequence context *in vitro*

**Figure 2.**
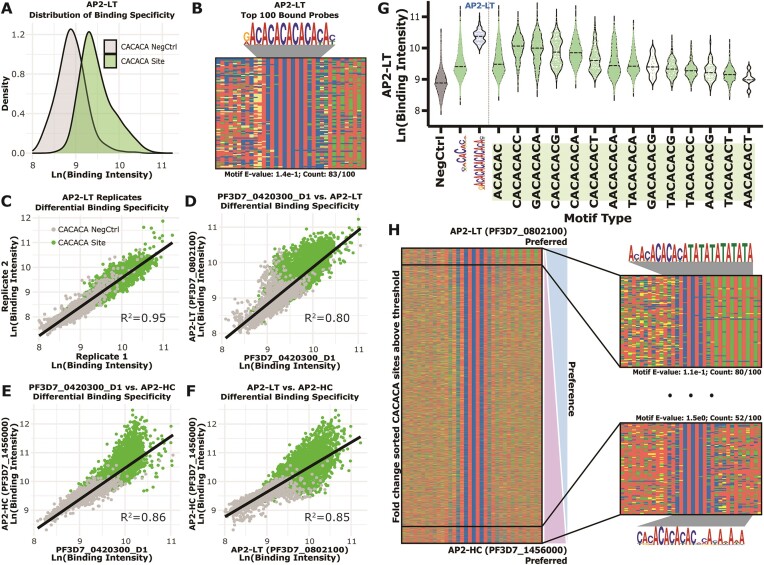
CACACA-binding AP2 domains have moderate differences in sequence context preferences at medium-to-low affinities. (**A**) Binding intensity distributions for CACACA probes and respective negative control probes for AP2-LT (CACACA probes [Green] and negative control probes [Grey]). Significantly different binding defined using a two-tailed Mann–Whitney test [*P*-value < 0.0001]); (**B**) four-color plot of the top 100 bound probes by AP2-LT with enriched motif above and calculated *E*-value and number of occurrences below. Color representations: A (red), C (blue), G (yellow) and T (green); (**C**) comparison of the gcPBM binding intensities for AP2-LT technical replicates (Pearson correlation: *R*^2^= 0.95) (CACACA probes [green] and negative control probes [grey]); (**D**) comparison of the gcPBM results for PF3D7_0420300_D1 versus AP2-LT (Pearson: *R*^2^= 0.80); (**E**) comparison of the gcPBM results for PF3D7_0420300_D1 versus AP2-HC (Pearson: *R*^2^= 0.86); (**F**) comparison of the gcPBM results for AP2-LT versus AP2-HC (Pearson: *R*^2^= 0.85); (**G**) binding intensity distributions from AP2-LT for negative control probes (grey), all CACACA probes (green), the AP2-LT extended motif probes (blue) and all 8-mer CACACA probes represented in the gcPBM (green; right of the vertical line). Dotted lines in each violin plot are the calculated mean; (**H**) *Left:* four-color plot of all CACACA probes above the threshold (defined by the 90th percentile of negative control probes) sorted by fold change (log_2_[AP2-LT/AP2-HC]). *Right:* zoom in on the top 100 differentially bound sites by AP2-LT (*top right*) and AP2-HC (*bottom right*) with enriched motifs, calculated *E*-values, and motif occurrence counts within those top 100 sites.

We first used gcPBM experiments to directly test if there were divergent sequence preferences for binding to the CACACA motif under different genomic contexts. Recombinantly expressed and purified DBDs were each run on the *P. falciparum* gcPBM in addition to a technical replicate of a representative DBD per motif type (i.e. AP2-LT DBD for the CACACA group) (Figure 2). Due to differences in binding intensities across probe orientations (Supplementary Figure S2B-M) we selected the highest natural log median binding intensity value from either the 5′ or 3′ orientation for each unique DNA probe. As expected, each AP2 DBD from the CACACA-binding group demonstrated a significant preference for CACACA probes over associated negative control probes (Figure 2A; Supplementary Figure S3A–C). For each DBD, the 100 probes bound at the highest gcPBM binding intensities showed a strong preference towards long CA-dinucleotide repeats, with a slightly degenerate AT-dinucleotide repeat in the 3′ flanks (Figure 2B, Supplementary Figure S3A–C). Compared to the AP2-LT technical replicate experiment (R^2^ = 0.95) (Figure 2C), individual pairwise comparisons of the binding intensities between all three of the paralogous CACACA-binding DBDs showed only moderate differences in DNA sequence preference: *R*^2^ = 0.80 (PF3D7_0420300_D1 versus AP2-LT), *R*^2^ = 0.86 (PF3D7_0420300_D1 versus AP2-HC), and *R*^2^ = 0.85 (AP2-LT versus AP2-HC), respectively (Figure 2D–F). These similarities in high affinity sites, along with the modest differences in sequence context preferences, suggested that sequence context had limited importance at high affinity sites and likely diverge at lower affinity binding.

To determine whether the high prevalence of longer CA-dinucleotide repeats was due to true binding preference, or the long CA-repeats created multiple binding events per DNA probe, we used electrophoretic mobility shift assays (EMSAs). Systematic mutation of the CA-dinucleotide repeats across a representative high affinity CACACA probe abolished the slower/higher-mobility shift (multimeric binding of AP2-LT DBDs) upon mutation of the two central CA-dinucleotide repeats (Supplementary Figure S4A). This suggests that many of the AP2-LT high intensity gcPBM probes with more than three CA-dinucleotide repeats likely resulted from an interaction between more than one AP2-LT DBD per DNA probe. Unlike the AP2-LT DBD, EMSAs with the same DNA probes using the AP2-HC DBD demonstrated a 1:1 (DBD:DNA) stoichiometry (Supplementary Figure S4A). Additional EMSA-based validation using AP2-LT and gcPBM dsDNA probes with low-, medium-, and high-affinities from both CACACA and negative control probes recapitulated the varying degrees of binding specificities shown in the high-throughput gcPBM experiments (Supplementary Figure S4B). Overall, these findings demonstrate that DNA-binding of the CACACA-binding ApiAP2 DBDs is not greatly impacted by sequence context and these proteins differ in their abilities to multimerize on DNA *in vitro*.

Due to the multimeric binding to high affinity probes by AP2-LT, we expanded our analysis to include all CACACA probes. This allowed us to further explore preferences for nucleotides directly adjacent to the central CACACA motif across a range of affinities. To categorize each protein-DNA binding event into specifically bound versus non-specifically bound, we set a threshold at the 90th percentile of the binding signal for negative control probes, as per previous studies (10,12). After parsing out the binding intensities for each 8-mer sequence represented on the gcPBM with the ‘ACACAC’ as the most represented central 6-mer (Supplemental File 5), we found that the central 8-mer sequences with the highest average binding signal were all extensions of the CA-dinucleotide repeat (Figure 2G; Supplementary Figure S5). However, calculating the fold change of the binding intensities between each pairwise comparison showed a strong preference for a short CA-repeat with flanking AT-dinucleotide repeats for AP2-LT, extended CA-repeats (up to six CA-dinucleotide repeats) for AP2-HC, and short CA-repeats with no flanking pattern for PF3D7_0420300_D1 (Figure 2H; Supplementary Figure S6). These differences in sequence context at lower affinity probes suggest that the modest differences in DNA binding resulted from medium-to-low affinity range interactions. Therefore, DNA sequence context may not play a large role in differential binding of CACACA-binding TFs *in vitro* suggesting that other factors must influence binding site selection *in vivo*.

### CACACA-binding transcription factors occupy differential sites *in vivo*

**Figure 3.**
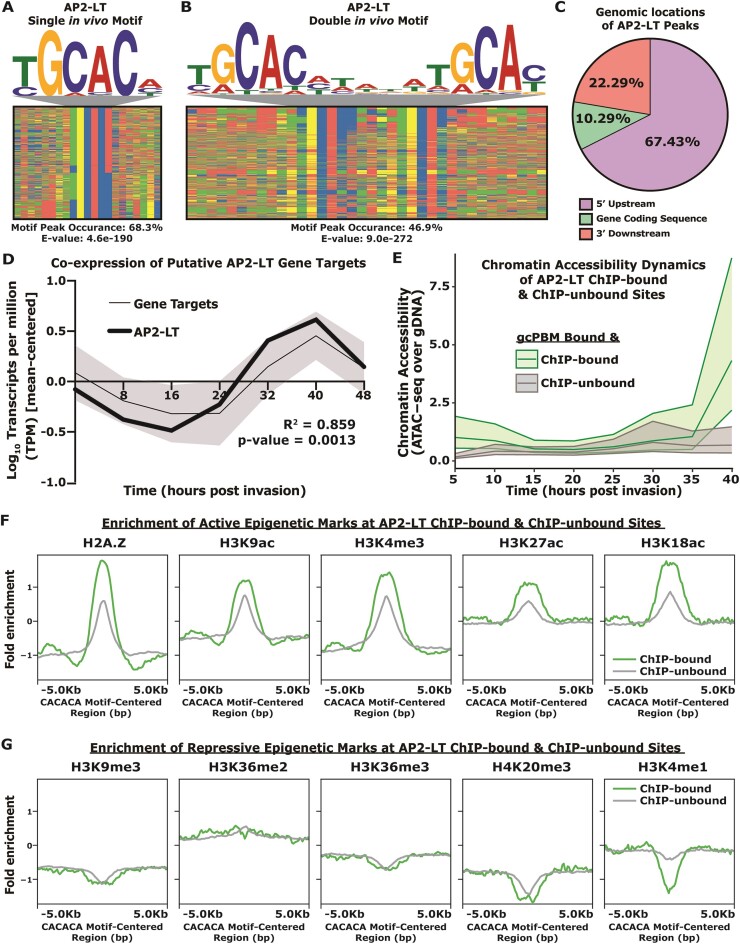
AP2-LT mostly binds to intergenic regions upstream of late-stage genes demarcated by chromatin accessibility and active epigenetic modifications *in vivo*. (**A**) Four-color plot of TGCAC-centered AP2-LT bound sites, enriched DNA motif above, calculated motif peak occurrence, and calculated motif *E*-value below. Color representations: A (red), C (blue), G (yellow) and T (green); (**B**) four-color plot of TGCACN_5_TGCAC-centered AP2-LT bound sites, enriched DNA motif above, calculated motif peak occurrence and calculated motif *E*-value below; (**C**) percent of MACS2-called peaks that overlap with 5′-upstream regions (purple), gene coding sequences (green), or 3′- downstream regions (peach); (**D**) mean-centered transcript abundance profile of 275 AP2-LT gene targets (mean [black] with one standard deviation [grey]) compared to the mean-centered transcript abundance profile of the AP2-LT transcript (bold green). Calculated *R*^2^ and *P*-value from Pearson correlation between putative gene targets and AP2-LT profiles bottom right; (**E**) chromatin accessibility across eight asexual stage timepoints (5 hpi, 10 hpi, 15 hpi, 20 hpi, 25 hpi, 30 hpi, 35 hpi and 40 hpi) for AP2-LT ChIP-bound (green) and ChIP-unbound (grey) sites. Central line plotted is the median normalized read count over gDNA control. Upper and lower lines are the 75th percentile and 25th percentile, respectively; (**F**) profile plot of the mean ChIP-seq fold enrichment (Log_2_[IP/Input]) of five active epigenetic marks (H2A.Z, H3K9ac, H3K4me3, H3K27ac and H3K18ac) for ChIP-bound (green) and ChIP-unbound (grey) sites; and (**G**) profile plot of the mean ChIP-seq fold enrichment (Log_2_[IP/Input]) of five repressive epigenetic marks (H3K9me3, H3K36me2/3, H4K20me3 and H3K4me1) for ChIP-bound (green) and ChIP-unbound (grey) sites.

Since the binding specificity of the CACACA-binding DBDs were not greatly impacted by sequence context, we investigated the potential impact of the *in vivo* chromatin landscape on genome-wide binding site selection. While AP2-HC has been fully characterized *in vivo* (85), the other CACACA-binding TFs have not. Therefore, we determined the *in vivo* genome-wide occupancy of AP2-LT using chromatin immunoprecipitation followed by sequencing (ChIP-seq) from *P. falciparum* cell culture. Three biological replicates of ChIP-seq using a CRISPR-modified clonal parasite line of AP2-LT^HA^ (Supplementary Figure S7) and a negative control sample were conducted at peak AP2-LT protein expression (36-45 h post invasion [hpi]) (87) (Supplementary Figure S8). Motif analysis of the ChIP-seq binding sites resulted in multiple highly-ranked motif variations of the *in vitro* CACACA motif (Supplementary Figure S9A). Surprisingly, the most enriched DNA sequence motif was TGCAC (E-value = 4.6e-190) (Figure 3A), in addition to a longer TGCACN_5_TGCAC motif (*E*-value = 9.0e-272) (Figure 3B; Supplementary Figure S9B-E). This fifteen nucleotide long, bipartite motif encompasses a full turn of the DNA helix, suggesting potential AP2-LT dimerization as we found via EMSA (Supplementary Figure S4B). Our ChIP-seq data indicated that AP2-LT mostly occupies regions upstream of transcription start sites (TSSs) as well as some gene coding sequences (Figure 3C; Supplemental File 4). Putative target genes (Supplemental File 6) were predicted based on the presence of a ChIP-seq peak no more than 2kb upstream of a target gene TSS (82) or within the gene coding sequence. The transcript abundance profiles for predicted target genes revealed co-expression with AP2-LT (Figure 3D) and GO terms (76) associated with parasite egress/invasion, protein modifications, and cell cycle (Supplemental File 7). These findings are consistent with previous reports that suggest that AP2-LT functions as an activator through interactions with the PfSAGA co-activator complex (83,84).

While many of the highly-ranked ChIP-seq DNA motifs were consistent with the *in vitro* CACACA motif, the top AP2-LT *in vivo*-bound TGCAC DNA motif is quite different than the *in vitro*-bound CACACA motif and resembles the GTGCAC motif bound by SIP2_D1, AP2-I_D3, and HDP1. We therefore hypothesize that AP2-LT may localize to sites enriched with TGCAC through cooperative interactions with GTGCAC-binding TFs or specific features of the nuclear environment may contribute to the divergent *in vivo* sequence specificity for AP2-LT. Additionally, we find that AP2-LT and AP2-HC bind mutually exclusive genome-wide binding sites *in vivo* (Supplementary Figure S10A) with AP2-LT in euchromatic regions and AP2-HC mostly localizing to heterochromatin via interactions with the heterochromatin protein1 (HP1) (85). Therefore, AP2-LT and AP2-HC do not occupy the same sites *in vivo* likely due to the influence of the nuclear environment, including the capacity to multimerize and interact with other co-factors.

### Chromatin accessibility and histone modifications differentiate *in vivo* binding site selection of CACACA-binding transcription factors

To further probe whether CACACA sequence context impacts TF binding site selection in the context of the nuclear environment, we compared genomic sequences bound by gcPBM with genomic sites bound via ChIP-seq. The gcPBM data was first grouped into gcPBM-bound and gcPBM-unbound probes using a threshold set at the 90th percentile of the CACACA negative control probes, and gcPBM-unbound were excluded as ‘non-specific binding’. The gcPBM-bound probes were further grouped into ChIP-bound versus ChIP-unbound sites using a minimum overlap of 21-bp of the 36-bp gcPBM sequences to ensure that the overlap contained the central DNA motif (Supplemental File 4). Of note, this analysis was not limited to ChIP-seq peaks that only contain the motif-of-interest, and included ChIP-seq peaks without the consensus motif in the ChIP-bound group. Interestingly, AP2-LT ChIP-bound sites were bound at significantly higher gcPBM binding intensities than ChIP-unbound sites. This is in contrast to the few AP2-HC ChIP-bound sites that were bound significantly lower than ChIP-unbound sites (Supplementary Figure S11A,B). These results demonstrate that binding site selection *in vivo* does not correlate with higher affinity binding *in vitro* for AP2-LT and AP2-HC.

The predicted DNA motifs for the ChIP-bound and ChIP-unbound sites also showed that the longer CA-dinucleotide repeats and flanking AT-repeats found by gcPBM (Figure 2H) were not enriched in the AP2-LT ChIP-bound sites (Supplementary Figure S11A). This further suggests a shift in sequence preference between *in vitro* and *in vivo* binding for AP2-LT. Additionally, we found that the change from a CACACA preference *in vitro* to TGCAC *in vivo* by AP2-LT (Supplementary Figure S12A,B) resulted in a low Pearson correlation between *in vitro* and *in vivo* binding for AP2-LT (Supplementary Figure S12C). After reanalyzing the published AP2-HC ChIP-seq (85) data, several euchromatic peaks contained the CACACA motif although it was not enriched overall (Supplementary Figure S11B) and AP2-HC data also resulted in a low Pearson correlation between *in vitro* and *in vivo* binding (Supplementary Figure S12D). These findings further suggest that *in vitro-*defined sequence context preferences are not the main drivers of *in vivo* binding site selection for AP2-LT and AP2-HC.

To explore the possible contribution of the chromatin landscape on TF binding, we compared the ChIP-bound and ChIP-unbound sites with published temporal genome-wide chromatin accessibility and epigenetic post-translational modification (PTM) datasets (47,71–73). For this analysis, we used the ChIP-unbound sites as a control to observe if changes in chromatin accessibility and epigenetic patterns were unique to the ChIP-bound sites. Using reanalyzed Assay for Transposase-Accessible Chromatin with sequencing (ATAC-seq) data (47) from eight timepoints, we found that ChIP-unbound sites were largely in inaccessible chromatin regions throughout the 48-h cycle (Figure 3E; Supplementary Figure S13). More interestingly, a dynamic opening of chromatin was observed at the ChIP-bound sites only during late-stage development, which coincides with the maximal expression of AP2-LT (87) (Figure 3E; Supplementary Figure S13). In contrast, AP2-HC associates with regions of inaccessible chromatin throughout parasite development, as expected (Supplementary Figure S11B).

We next determined whether ChIP-bound and ChIP-unbound sites were differentially demarcated by histone activation or repression marks (71–73) when AP2-LT and AP2-HC are maximally expressed (87). We found that activation marks (H2A.Z, H3K9ac, H3K4me3, H3K27ac and H3K18ac) were highly enriched at AP2-LT ChIP-bound sites, while the repression marks (H3K9me3, H3K36me2/3, H4K20me3 and H3K4me1) were not (Figure 3F,G; Supplementary Figure S14). In contrast, AP2-HC ChIP-bound sites were enriched with repressive marks at binding sites, with the most represented mark being heterochromatic H3K9me3 (Supplementary Figure S15). We only found a modest difference in epigenetic mark enrichment between the ChIP-bound versus ChIP-unbound sites, which suggests that these epigenetic marks co-occur at ChIP-bound sites, but do not define the binding site selection. Overall, these results indicate that genome-wide binding by AP2-LT and AP2-HC correlates with chromatin state.

### Non-paralogous GTGCAC-binding transcription factors have distinct sequence context preferences by gcPBM

**Figure 4.**
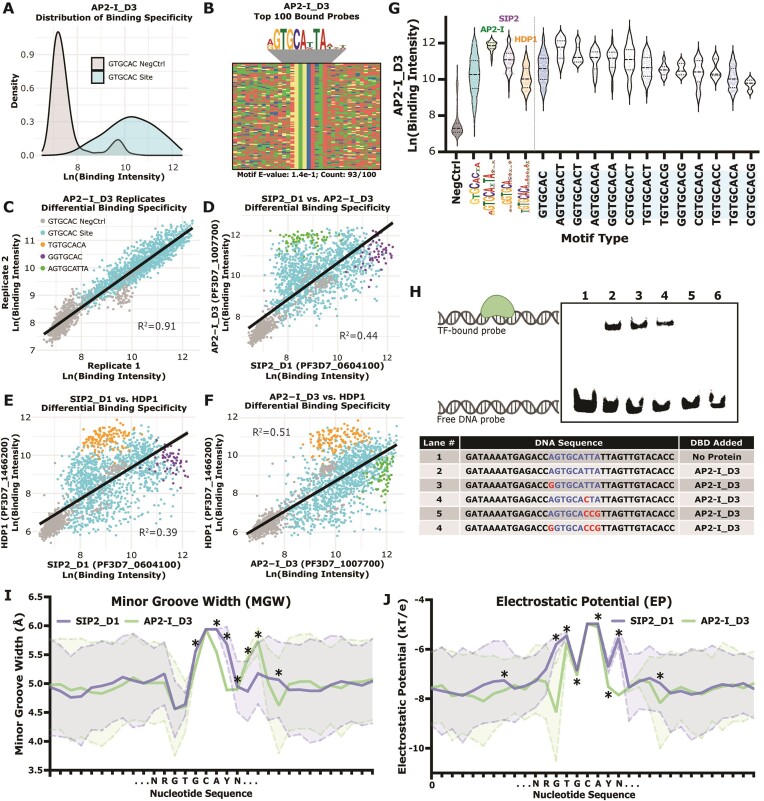
Binding specificity is dependent on nucleotides proximal to the GTGCAC motif. (**A**) Binding intensity distributions for GTGCAC probes and the respective negative control probes for AP2-I_D3. (GTGCAC probes [blue] and negative control probes [grey]). Significantly different binding defined using a two-tailed Mann–Whitney test [*P*-value < 0.0001]); (**B**) four-color plot of top 100 bound probes by AP2-I_D3 with enriched motif above and calculated *E*-value and number of occurrences below. Color representations: A (red), C (blue), G (yellow) and T (green); (**C**) comparison of the binding intensities for AP2-I_D3 technical replicates (Pearson correlation: *R*^2^= 0.912). (Negative control probes [grey], GTGCAC probes [blue], HDP1-preferred TGTGCACA probes [orange], SIP2_D1-preferred GGTGCAC probes [purple], and AP2-I_D3-preferred AGTGCATTA probes [green]; (**D**) comparison between SIP2_D1 and AP2-I_D3 (Pearson: *R*^2^= 0.442). (**E**) Comparison between SIP2_D1 and HDP1 (Pearson: *R*^2^= 0.386). (**F**) Comparison between AP2-I_D3 and HDP1 (Pearson: *R*^2^= 0.514); (**G**) binding intensity distributions from AP2-I_D3 for GTGCAC negative control probes (grey), all GTGCAC probes (blue), the extended motif probes by all three GTGCAC-binding TFs (AP2-I_D3 [green], SIP2_D1 [purple] and HDP1 [orange]), and 8-mer GTGCAC probes represented in the gcPBM (blue; right of the vertical line); (**H**) EMSA of AP2-I_D3 binding to a AGTGCATTA probe with increasing numbers of mutations to the extended motif. Protein–DNA interaction graphic was created with BioRender.com; (**I**) calculated minor groove width (MGW) predictions across all AGTGCATTA probes (green) and all GGTGCAC probes (purple). Solid line represents the mean and dotted lines encompassing the shaded area is one standard deviation. *Denotes statistically significant differences (*P*-value < 0.05) between means (two-sided Wilcoxon rank sum test). N = IUPAC for any nucleotide. Y = IUPAC for C or T nucleotides; (**J**) calculated electrostatic potential (EP) predictions across all AGTGCATTA probes (green) and all GGTGCAC probes (purple). Solid line represents the mean and dotted lines encompassing the shaded area is one standard deviation. *Denotes statistically significant differences (*P*-value < 0.05) between means (two-sided Wilcoxon rank sum test). N = IUPAC for any nucleotide. Y = IUPAC for C or T nucleotides.

To investigate binding site selection for a set of non-paralogous TFs, we used our *P. falciparum* gcPBM and the DBDs from three TFs that bind a GTGCAC motif *in vitro*. These include two ApiAP2 proteins, domain one [D1] from SIP2 and domain three [D3] from AP2-I, and a homeodomain from HDP1 (Figure 1A). All three DBDs showed a binding preference for GTGCAC probes over negative control probes (Figure 4A; Supplementary Figure S3D-F). The distribution of binding to GTGCAC probes was relatively broader, indicating a greater impact of sequence context on DNA binding (Figure 4A; Supplementary Figure S3D-F). Additionally, the top 100 GTGCAC probes revealed an extended sequence preference beyond the core GTGCAC motif (41,48) of GGTGCAC for SIP2_D1, AGTGCATTA for AP2-I_D3 and TGTGCACA for HDP1 (Figure 4B; Supplementary Figure S3D–F).

Compared to strong correlation between AP2-I_D3 gcPBM technical replicates (*R*^2^ = 0.91; Figure 4C), pairwise comparisons of the gcPBM data for the GTGCAC DBDs demonstrated pronounced differences in DNA sequence context preferences (Figure 4D-F) with low Pearson correlations: *R*^2^ = 0.44 (SIP2_D1 versus AP2-I_D3), *R*^2^ = 0.39 (SIP2_D1 versus HDP1) and *R*^2^ = 0.51 (AP2-I_D3 versus HDP1), respectively (Figure 4D–F). To identify sequence preferences proximal to the core GTGCAC motif, we analyzed the binding intensity for each GTGCAC-binding DBD across the three extended motifs (AP2-I_D3: AGTGCATTA, SIP2_D1: GGTGCAC and HDP1: TGTGCACA) (Figure 4G; Supplementary Figure S16). We found preferences for additional single nucleotides proximal to the GTGCAC core sequence by parsing out the binding intensities for all 8-mers with central ‘GTGCAC’ sequences represented on the gcPBM (Supplemental File 5). These findings further indicated an influence of sequence context on TF binding *in vitro* for the GTGCAC-binding TFs (Figure 4G; Supplementary Figure S16 and S17).

By EMSA, we tested whether the core GTGCAC motif was sufficient for DNA binding or whether the extended motif bound by AP2-I_D3 was necessary (Figure 4H). To do this, we used a representative 36-bp sequence with the following properties: contained the central AGTGCATTA extended motif, was bound at a high affinity in the *in vitro* gcPBM, and was located in an *in vivo* AP2-I ChIP-seq bound region (79,90). Increased mutations to the extended nucleotides flanking the core GTGCAC 6-mer motif resulted a reduction in AP2-I_D3 binding, suggesting that the core sequence context is indeed important for binding (Figure 4H). Overall, these results indicate that the sequence context of the GTGCAC motif greatly influences differential binding between the of SIP2_D1, AP2-I_D3 and HDP1 DBDs.

### GTGCAC-binding transcription factors prefer DNA sequences with diverse predicted DNA shapes

We noted that specific nucleotide patterns distal to the GTGCAC motif (more than three nucleotides upstream/downstream of the extended motif) were not enriched in the probes preferred by the GTGCAC-binding factors (Supplementary Figure S17). This is in stark contrast to what we observed for the CACACA-binding factors (Figure 2H; Supplementary Figure S6). Therefore, we determined the contribution of DNA shape readout of sequence-dependent DNA topologies on TF binding in addition to base readout mechanisms (5). These DNA shape measurements take into consideration how the local flexibility or intrinsic shape of the DNA impacts docking of a TF into its preferred DNA motif, including the effects of 2- to 5-mer nucleotide patterns via inter- and intra-nucleotide interactions. While there are numerous DNA shape features (5), we focused on minor groove width (MGW) and electrostatic potential (EP), which are highly predictive of DNA-binding specificity (77,94–96). Using DNAshapeR (78), we predicted MGW and EP profiles for all DNA probes containing the AGTGCATTA, GGTGCAC, and TGTGCACA extended motifs. Significant differences between each pairwise comparison for MGW and EP suggested that, in addition to base-specific contacts, DNA shape-readout mechanisms likely influence *in vitro* binding specificity of the GTGCAC-binding DBDs (Figure 4I, J; Supplementary Figure S18A–D).

To determine the impact of our predicted contribution of DNA shape on binding, the AP2-I_D3:DNA interaction was tested by EMSA using specific DNA mutations that maximize the change of predicted MGW and EP, while minimizing the number of mutated nucleotides (Supplemental File 3). Distal shape mutations (more than three nucleotides upstream/downstream of the extended AGTGCATTA motif) (Supplementary Figure S19) did not impact the strength of the AP2-I_D3:DNA-bound state (Supplementary Figure S18E), suggesting that specific DNA shapes of the distal flanking sequences are not required for AP2-I_D3 to bind a high-affinity site. Therefore, we conclude that DNA sequence/shape context of proximal, but not distal, nucleotides relative to the GTGCAC motif contribute to differential specificity for the GTGCAC-binding DBDs.

### Sequence context, chromatin state, and timing of expression differentiate *in vivo* binding site selection of GTGCAC-binding transcription factors

As demonstrated by the gcPBM binding results (Figure 4), DNA sequence context and intrinsic DNA shape can impact the *in vitro* binding of the GTGCAC group (SIP2_D1, AP2-I_D3, and HDP1). To explore the impact of sequence context on binding site selection *in vivo*, we compared the *in vitro* gcPBM binding intensities to the available *in vivo* ChIP-seq genome-wide occupancy data for AP2-I (79) and HDP1 (48) which only shared 47 binding sites (Supplementary Figure S10B). Genomic sites bound by AP2-I *in vivo* also revealed an enrichment for the extended AGTGCATTA motif (Supplementary Figure S11C), with moderate correlation between *in vitro* and *in vivo* binding (Supplementary Figure S20A-C). Chromatin accessibility (47) and epigenetic PTMs (71–73) at AP2-I ChIP-bound and ChIP-unbound sites showed that ChIP-bound sites were almost exclusively accessible (Supplementary Figure S11C) and enriched with activation marks (Supplementary Figure S21), in contrast to ChIP-unbound sites, suggesting that AP2-I requires sites of open and active euchromatin. A reanalysis of HDP1 ChIP-seq data identified genome-wide binding to intergenic regions upstream of genes during the sexual blood stage development (48). We found that the extended TGTGCACA motif preferred by HDP1 *in vitro* (Supplementary Figure S3F) is also enriched *in vivo* (Supplementary Figure S11D), although there was low correlation between the *in vitro* and *in vivo* bound sites (Supplementary Figure S20D-F). We conclude that distinct *in vivo* binding site selection is largely due to differences in the extended DNA motifs and timing of expression in the parasite lifecycle.

### Low affinity DNA-binding preferences across divergent DNA motifs may influence TF *in vivo* genome-wide co-occupancy

Based on comparisons of published *in vivo* binding site data from ChIP-seq studies, AP2-G and AP2-EXP recognize unique DNA motifs, yet both co-occupy a subset of overlapping genomic regions with AP2-I (79,92). Because the *P. falciparum*-specific gcPBM design contains all four motifs types (CACACA, GTGCAC, GTAC and TGCATGCA) (Figure 1B) this allowed us to re-examine our data to interrogate TF binding at other enriched DNA motifs represented on the Pf gcPBM (79,86,92). To characterize possible mechanisms of co-occupancy between AP2-G and AP2-I we first determined the genome-wide binding specificity of AP2-G using the *P. falciparum*-specific gcPBM. AP2-G bound to GTAC probes with a narrow distribution of binding and high correlation between technical replicates (Supplementary Figure S22A-D), which implied a low importance for sequence context. While the AP2-G ChIP-bound sites resulted in higher binding intensities over all other genomic GTAC-containing sites (Supplementary Figure S11E), the *in vitro* gcPBM versus *in vivo* ChIP-seq binding were only moderately correlated (Supplementary Figure S23A).

**Figure 5.**
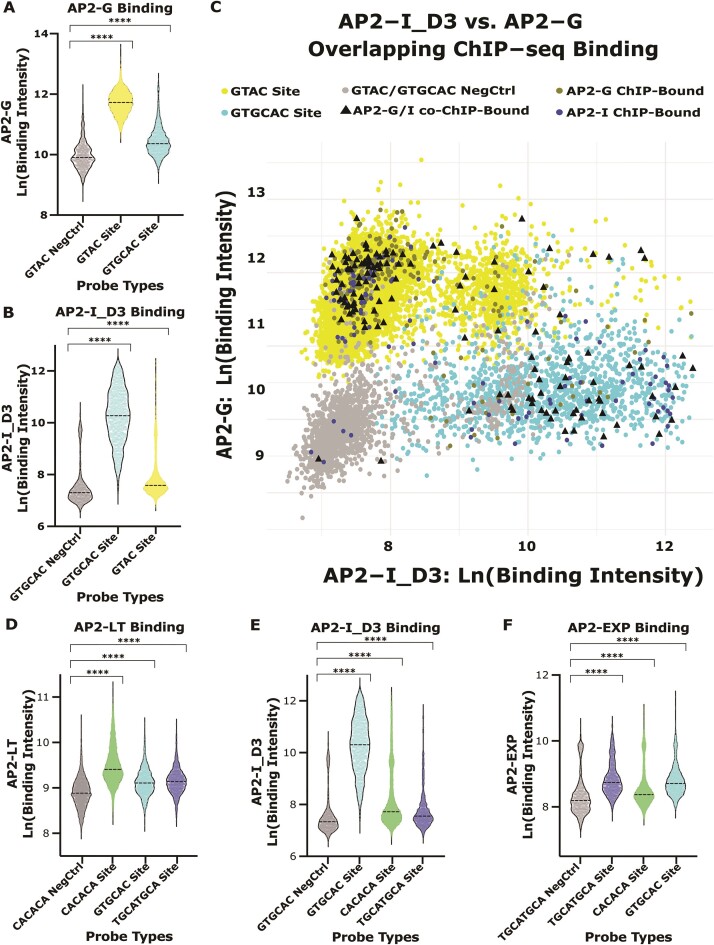
Overlapping *in vitro* binding preferences across DNA motif types. (**A**) AP2-G binding intensity distributions for GTAC negative control probes (grey), all GTAC probes (yellow) and all GTGCAC probes (blue); (**B**) AP2-I_D3 binding intensity distributions for GTGCAC negative control probes (grey), all GTGCAC probes (blue) and all GTAC probes (yellow); (**C**) comparison of the binding intensities for AP2-G and AP2-I_D3. Negative control probes (grey), GTAC probes (yellow), GTGCAC probes (blue), AP2-G ChIP-bound sites (dark yellow), AP2-I ChIP-bound sites (dark blue) and AP2-G/AP2-I co-bound ChIP-bound sites (black triangles); (**D**) AP2-LT binding intensity distributions for CACACA negative control probes (grey), all CACACA probes (green), all GTGCAC probes (blue) and all TGCATGCA probes (purple); (**E**) AP2-I_D3 binding intensity distributions for GTGCAC negative control probes (grey), all GTGCAC probes (blue), all CACACA probes (green), and all TGCATGCA probes (purple); (**F**) AP2-EXP binding intensity distributions for TGCATGCA negative control probes (grey), all TGCATGCA probes (purple), all GTGCAC probes (blue), and all CACACA probes (green).

AP2-G and AP2-I recognize divergent DNA motifs *in vitro* (Figure 1A) although roughly one-third of *in vivo* ChIP-seq binding sites are shared between these TFs (Supplementary Figure S10C). When we analyzed the AP2-G and AP2-I_D3 gcPBM, we found that AP2-G bound GTGCAC probes significantly above GTAC negative control probes (Figure 5A) and AP2-I_D3 bound GTAC probes significantly above GTGCAC negative control sites (Figure 5B) at low affinities, suggesting that these DBDs also bind divergent DNA motifs *in vitro*. To determine if these lower affinity sites are also bound *in vivo* by both AP2-G and AP2-I, we next compared the AP2-G and AP2-I_D3 ChIP-bound sites to categorize sites that were co-occupied by both TFs (‘co-ChIP-bound’). By calculating the fold change of gcPBM binding between the co-ChIP-bound sites and grouping the data as: preferred by AP2-I (Log_2_FC > 2), preferred by AP2-G (Log_2_FC > –2), or equally preferred by both DBDs *in vitro* (2 > Log_2_FC > –2) (Figure 5C), we indeed find that they bound co-occupied sites at low affinities as observed *in vivo*.

Similarly, our AP2-LT ChIP-seq results identified an overlap with some AP2-EXP genome-wide binding sites, and previous work reported an overlap of AP2-I and AP2-EXP *in vivo* binding site preference (92). Therefore, to expand the comparison to include all three factors, we identified the overlapping ChIP-seq peaks between AP2-LT (CACACA-binder), AP2-I (GTGCAC-binder) and AP2-EXP (TGCATGCA-binder) (Supplementary Figure S10D). For this comparison, we first identified the genome-wide binding specificity for AP2-EXP using the *P. falciparum*-specific gcPBM. We found that AP2-EXP bound to TGCATGCA probes at higher signal intensities with a strong correlation between technical replicates (Supplementary Figure S22E–H). The gcPBM signal intensity of AP2-EXP ChIP-bound sites compared to all TGCATGCA sites had no significant difference (Supplementary Figure S11F), and the *in vitro* versus *in vivo* binding comparison showed only a moderate-to-low correlation (Supplementary Figure S23B), which suggested no dependence on sequence context. AP2-EXP ChIP-bound sites were mostly defined by accessible chromatin (Supplementary Figure S11F) and activating marks (Supplementary Figure S24). We also analyzed the AP2-LT, AP2-I_D3, and AP2-EXP gcPBM data for binding to CACACA-, GTGCAC- and TGCATGCA-containing probes. Each pairwise comparison (AP2-LT versus AP2-I_D3, AP2-LT vs. AP2-EXP and AP2-I_D3 versus AP2-EXP) resulted in significant low affinity binding to probes containing other DNA motifs over control probes (Figure 5D–F), with a wide distribution of co-ChIP-bound sites (Supplementary Figure S25). Therefore, we conclude that some AP2 DBDs (AP2-G, AP2-I, AP2-LT and AP2-EXP) have the propensity to bind different DNA motifs *in vitro* at lower affinities which likely results in the co-occupancy observed by *in vivo* ChIP-seq binding data.

## Discussion

Transcription factor (TF) binding site recognition and specificity are important components of gene regulation in all living organisms (1,7,8,97–100). However, the short and conserved DNA motifs bound by a TF may not fully capture biologically relevant binding site preferences at lower affinity genomic sites (28). Moreover, features such as DNA sequence context, local DNA shape, protein-protein interaction partners, epigenetic post-translational modifications, and chromatin architecture also impact binding site recognition (5,8,11,28,97). Investigating the parameters that differentiate the sequence specificity of large families of paralogous TFs in model eukaryotes poses a great challenge since they are often numerous (>100) and functionally redundant (9). In this study, we explored the contribution of these context-dependent parameters in the malaria parasite *P. falciparum*, which has a reduced number of TFs relative to other eukaryotes (5,7,8,11,28).

Using a novel *P. falciparum* genomic-context protein-binding DNA microarray (gcPBM), we found that three ApiAP2 TFs with DBDs that recognize a CACACA motif (PF3D7_0420300_D1, AP2-LT and AP2-HC) cannot readily differentiate DNA sequence context *in vitro*. Despite this observation, we found that AP2-LT and AP2-HC (85) do not bind overlapping genomic regions *in vivo*. We correlated our findings with previously published co-factor interactions, chromatin accessibility, and epigenetic mark datasets (47,48,71–73,79,85,92) and found that chromatin and protein-complex features likely play important roles in defining the binding site selections *in vivo*. The absence of overlapping *in vivo* binding for AP2-LT and AP2-HC TFs is supported by recent studies that identified AP2-HC complexed with PfHP1 (85) at heterochromatic regions, while AP2-LT is a component of the putative PfSAGA transcriptional co-activating complex (84) which is only found in euchromatic regions. While the specific role of PF3D7_0420300 as a TF during *P. falciparum* asexual blood stage development remains unknown, a previous publication identified its interaction with PfMORC (101), a putative repressive complex component highly characterized in metazoans (102–109) and recently in *Toxoplasma gondii* (110,111). Therefore, we anticipate that a PF3D7_0420300:PfMORC complex would likely bind genomic regions not bound by AP2-LT or AP2-HC. We also find that AP2-LT binds a double (TG/CA)CAC motif repeated in an eleven-nucleotide pattern *in vitro* and *in vivo*, suggesting a potential requirement for AP2-LT dimerization at genomic targets. Although a co-crystal structure of AP2-EXP:DNA revealed the possibility for AP2 domain-swapped homodimerization (64), AP2-LT likely binds to the same face of the DNA via a different dimerization mechanism. In addition, we found that genomic sites bound by AP2-LT become accessible as the TF is being expressed at the mRNA (82) and protein (87) level, suggesting an interplay between TF occupancy and nucleosome positioning. Therefore, AP2-LT may recognize the TGCAC DNA motif on or near promoter-bound nucleosomes and recruit the PfSAGA chromatin remodelers (84) to increase DNA accessibility. This potential activity for AP2-LT is reminiscent of pioneer factors in other eukaryotes (112–115), but remains to be tested. We conclude that, while the three CACACA-binding TFs bound similar DNA sequence context specificities by gcPBM *in vitro*, most of the TFs had divergent chromatin preferences, suggesting that they are unlikely to be functionally redundant.

In contrast, our gcPBM results identified that SIP2_D1, AP2-I_D3 and HDP1, which bind the GTGCAC sequence, have differing *in vitro* preferences for nucleotides proximal to this core motif. These TFs also display a preference for divergent predicted DNA shape features, such as minor groove width and electrostatic potential, suggesting that shape-readout mechanisms (5) additionally impact binding site selection. Future structural work with these DBDs may help further define the base- and shape-readout mechanisms of these TF-DNA interactions. *In vivo* AP2-I prefers the AGTGCATTA extended motif and binds to regions of accessible chromatin containing activating epigenetic marks. SIP2 prefers a bipartite SPE2 GGTGCAC extended motif and colocalizes to sub-telomeric regions of inaccessible chromatin and repressive epigenetic marks (88). For AP2-I and SIP2, a subset of cells from single-cell transcriptomics data (70) show co-expression of both factors, suggesting the possibility of co-binding or competitive binding, which remains to be determined. Finally, HDP1 binds the extended TGTGCACA motif, yet is maximally expressed during sexual blood stages, for which comprehensive chromatin accessibility and epigenetic modifications datasets are limited. Overall, we found that the GTGCAC-binding TFs have differential *in vitro* and *in vivo* DNA-binding preferences due to contributions from DNA sequence/shape context preferences, timing of expression, chromatin accessibility, and epigenetic patterns.

Our results also suggest that low-affinity protein-DNA interactions by *P. falciparum* DBDs may contribute to a more complex mechanism of *in vivo* TF co-occupancy. This concept has been thoroughly investigated in the homeobox domain (HOX) TF family in *Drosophila*, where sites of Exd:Hox co-occupancies are driven by low-affinity binding between the TFs and DNA by latent specificity (11,28). In the malaria rodent model, *P. berghei*, recent work has also identified co-occupancy of ApiAP2 TFs and other TFs (116). Additionally, in *P. falciparum*, *in vivo* co-occupancy between AP2-G (GTAC-binder) and AP2-I (GTGCAC-binder) has been previously reported (79). Here we further identified a subset of co-occupied sites by AP2-LT (CACACA-binder), AP2-I_D3 (79,90), and AP2-EXP (92) (TGCATGCA-binder). It was previously hypothesized that, at sites of co-occupancy, one TF is driving the DNA-specific binding, while the other factor is present by protein-protein interactions (79). However, using gcPBM binding data for the above DBDs, we found that each DBD bound to divergent DNA motifs at low affinities, suggesting co-occupancy may be impacted by low-affinity binding from either factor. Overall, these results add to the growing evidence for genome-wide co-occupancy by ApiAP2 TFs. Future work on the physical interactions between *P. falciparum* TFs will further allow for the interrogation of putative cooperativity between these proteins.

This work contributes to our current understanding of how paralogous TF binding specificity is determined (44,46,56–58). By interrogating sequence and chromatin features using *Plasmodium falciparum* TFs we characterized a reduced set of paralogous DBDs from essential TFs with non-redundant functional roles. In this context, we found several solutions including TFs that rely on sequence context to differentiate genome-wide binding site selection and others that are coordinated through changes in chromatin state features. Our findings are also relevant from a therapeutic perspective since ApiAP2 TFs are unique to plant and eukaryotic parasite genomes and have therefore been proposed as future antiparasitic drug targets (92,117–121). Understanding the factors that drive complex gene regulatory mechanisms in *P. falciparum* is critical to selecting appropriate future drug interventions (122–124). Recent work has identified putative antimalarial compounds that interact with AP2 domains *in silico* and *in vitro* which arrest *Plasmodium* spp. development at multiple stages of the parasite life cycle (92). The concept of a ‘pan-ApiAP2 inhibitor’ design would allow for the inhibition of multiple ApiAP2 TFs, such as the group of CACACA-binding TFs that regulate divergent developmental pathways, with one drug. Understanding the specific roles of these unique and parasite-essential factors will be critical for the design of future AP2-targeted antimalarial therapies.

## Supplementary Material

gkae585_Supplemental_Files

## Data Availability

Data from genomic-context protein-binding microarray (gcPBM) experiments as raw and processed image and signal files is located on the NCBI Gene Expression Omnibus (GEO) [GSE227873]. Agilent ID generated by GEO [Agilent-085718] and placed as an online GEO platform. Whole genome sequencing data for AP2-LT^HA^ parasite line is located on the NCBI Sequence Read Archive (SRA) [PRJNA873081]. Genome-wide AP2-LT binding data from chromatin immunoprecipitation followed by sequencing (ChIP-seq) experiments is located as fully processed on NCBI Gene Expression Omnibus (GEO) [GSE212052]. ChIP-seq genome tracks (.bigwig) and unprocessed gel images are attached to the supplement and located on the Zenodo database [10.5281/zenodo.7007554].

## Notes

Present address: Yuning Zhang, Department of Genetics, Washington University School of Medicine in St. Louis, St. Louis, MO, 63110, USA.

Present address: Gabrielle A. Josling, Australian Red Cross Lifeblood, Melbourne, Victoria, 3003, Australia.
