# Correction to ‘DNA sequence and chromatin differentiate sequence-specific transcription factor binding in the human malaria parasite *Plasmodium falciparum*’

**DOI:** 10.1093/nar/gkae733

**Published:** 2024-08-24

This is a correction to: Victoria A Bonnell, Yuning Zhang, Alan S Brown, John Horton, Gabrielle A Josling, Tsu-Pei Chiu, Remo Rohs, Shaun Mahony, Raluca Gordân, Manuel Llinás, DNA sequence and chromatin differentiate sequence-specific transcription factor binding in the human malaria parasite *Plasmodium falciparum*, *Nucleic Acids Research*, 2024; gkae585, https://doi.org/10.1093/nar/gkae585

In the originally published version of this manuscript, errors were present in the institutions stated in affiliations 1, 2, 3, 4, 5 and 6.

Additionally, institutional information in affiliation 11 was incomplete.

The affected affiliations have been corrected to read as follows:

^1^Department of Biochemistry and Molecular Biology, The Pennsylvania State University, University Park, PA 16802, USA

^2^Huck Institutes Center for Eukaryotic Gene Regulation, The Pennsylvania State University, University Park, PA 16802, USA

^3^Huck Institutes Center for Malaria Research, The Pennsylvania State University, University Park, PA 16802, USA

^4^Center for Genomic and Computational Biology, Duke University, Durham, NC 27708, USA

^5^Department of Biostatistics and Bioinformatics, Duke University, Durham, NC 27708, USA

^6^Program in Computational Biology and Bioinformatics, Duke University, Durham, NC 27708, USA

^11^Department of Computer Science, Duke University, Durham, NC 27708, USA

