# Supplementary material for: DNA sequence and chromatin differentiate sequence-specific transcription factor binding in the human malaria parasite Plasmodium falciparum
Source: Nucleic Acids Res. 2024 Jul 5;52(17):10161–79. doi: 10.1093/nar/gkae585 (PMC11417369; doi:10.1093/nar/gkae585)
Supplement: gkae585_Supplemental_Files [file gkae585_supplemental_files.zip › Bonnell_NAR_Supplemental_FiguresWithCaptions_06_21_2024.docx]

**DNA sequence and chromatin differentiate sequence-specific transcription factor binding in the human malaria parasite *Plasmodium falciparum***

Victoria A. Bonnell^1,2,3^, Yuning Zhang^4,5,6^, Alan S. Brown Jr.^1,2,3^, John Horton^4,5^, Gabrielle A. Josling^1,2,3^, Tsu-Pei Chiu^7^, Remo Rohs^7,8,9,10^, Shaun Mahony^1,2^, Raluca Gordân^4,5,11,12^, & Manuel Llinás^1,2,3,13^

^1^Department of Biochemistry and Molecular Biology, ^2^Huck Institutes Center for Eukaryotic Gene Regulation, ^3^Huck Institutes Center for Malaria Research, ^13^Department of Chemistry, The Pennsylvania State University, University Park, PA 16802, United States

^4^Center for Genomic and Computational Biology, ^5^Department of Biostatistics and Bioinformatics, ^6^Program in Computational Biology and Bioinformatics, ^11^Department of Computer Science, ^12^Department of Molecular Genetics and Microbiology, Duke University, Durham, NC 27708, United States

^7^Department of Quantitative and Computational Biology, ^8^Department of Chemistry, ^9^Department of Physics and Astronomy, and ^10^Thomas Lord Department of Computer Science, University of Southern California, Los Angeles, CA 90089, United States

* To whom correspondence should be addressed. Tel: +1(814)867-3444; Email: manuel@psu.edu

**Supplemental Figures with captions:**


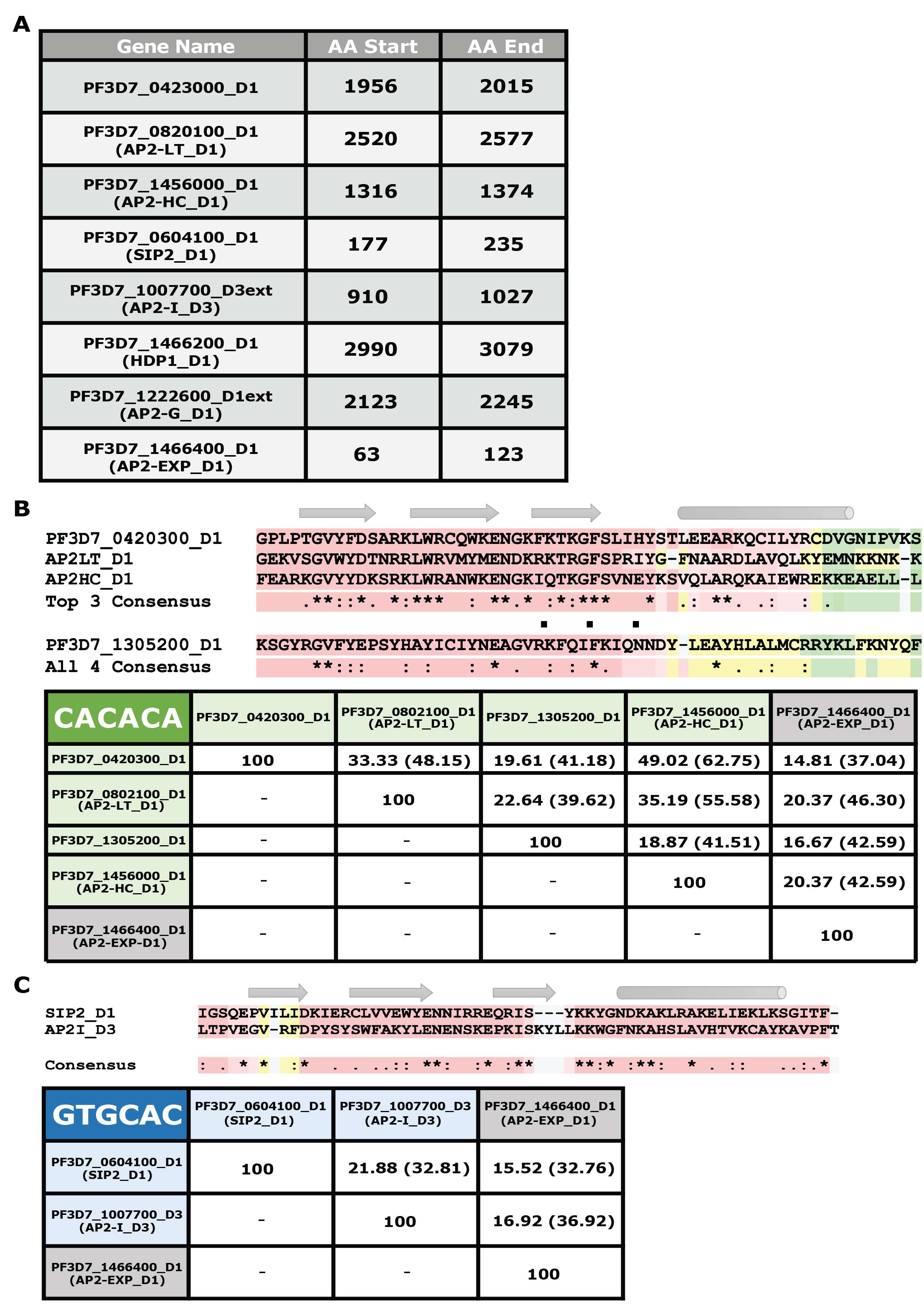


**Supplemental Figure 1: Amino acid sequence alignments of DBDs used in this study**

**(A)** Amino acid coordinates of DBDs used for gcPBM experiments; **(B)** *Above:* Amino acid sequence alignment of CACACA-binding AP2 domains. *Below:* Calculated percent identity (and percent similarity) from the CACACA-binding alignment with AP2-EXP as an outgroup. The CACACA-binding DBD from PF3D7_1305200 was excluded from this study as it was less conserved relative to the other CACACA-binding DBDs (20.37% identity) and is not co-expressed; **(C)** *Above:* Amino acid sequence alignment of GTGCAC-binding AP2 domains. *Below:* Calculated percent identity (and percent similarity) from the GTGCAC-binding alignment with AP2-EXP as an outgroup.


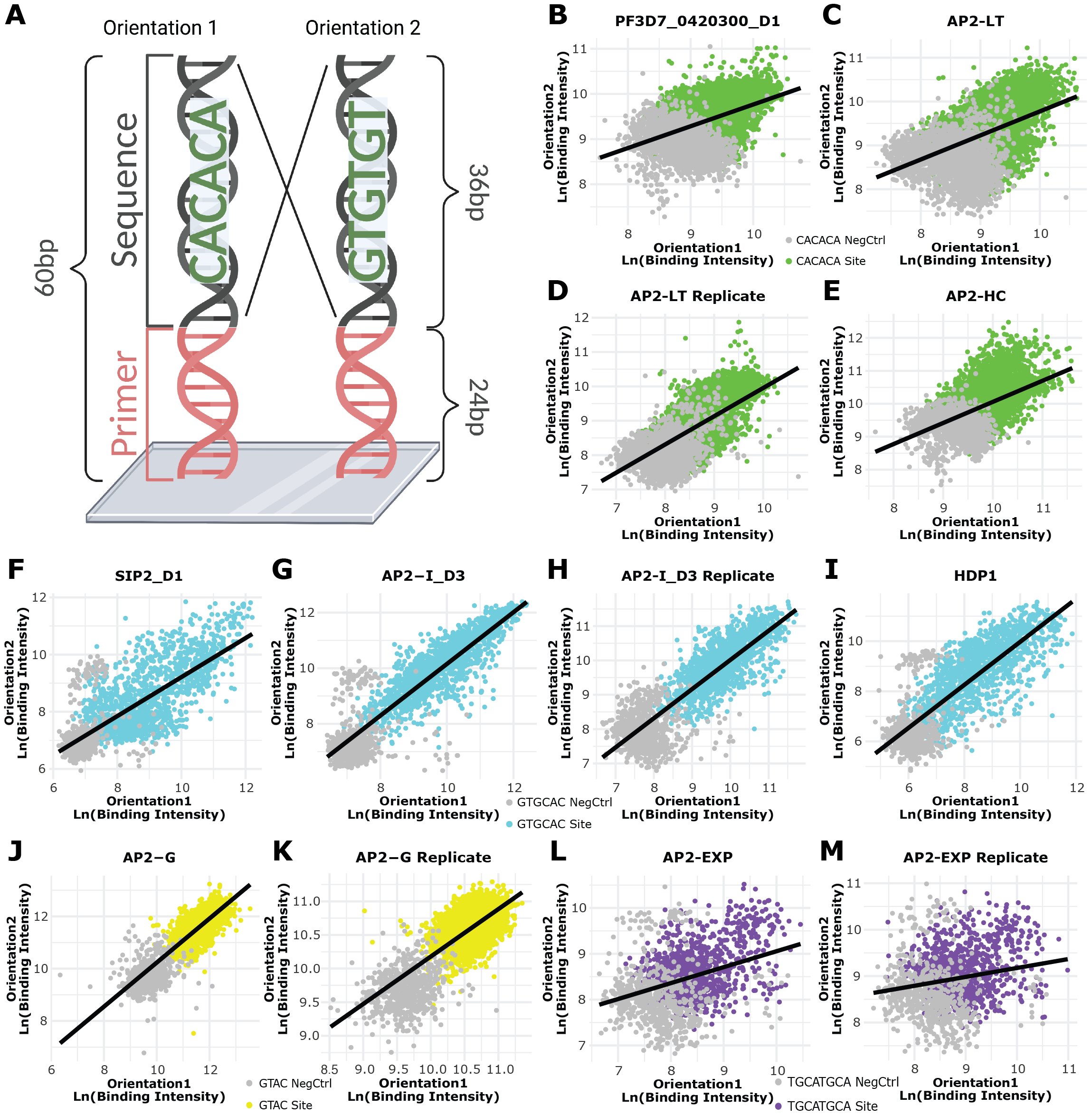


**Supplemental Figure 2: Differences in DNA-binding across probe orientations**

**(A)** Graphical representation of the two orientations from gcPBM experiments. Microarray graphic was created with BioRender.com; **(B-E)** Comparison of binding intensity across probe orientations for the CACACA-binding group including the technical replicate (CACACA probes [Green] and negative control probes [Grey]); **(F-I)** Comparison of binding intensity across probe orientations for the GTGCAC-binding group including the technical replicate (GTGCAC probes [Blue] and negative control probes [Grey]); **(J-K)** Comparison of binding intensity across probe orientations for AP2-G including the technical replicate (GTAC probes [Yellow] and negative control probes [Grey]); **(L-M)** Comparison of binding intensity across probe orientations for AP2-EXP including a technical replicate (TGCATGCA probes [Purple] and negative control probes [Grey]).


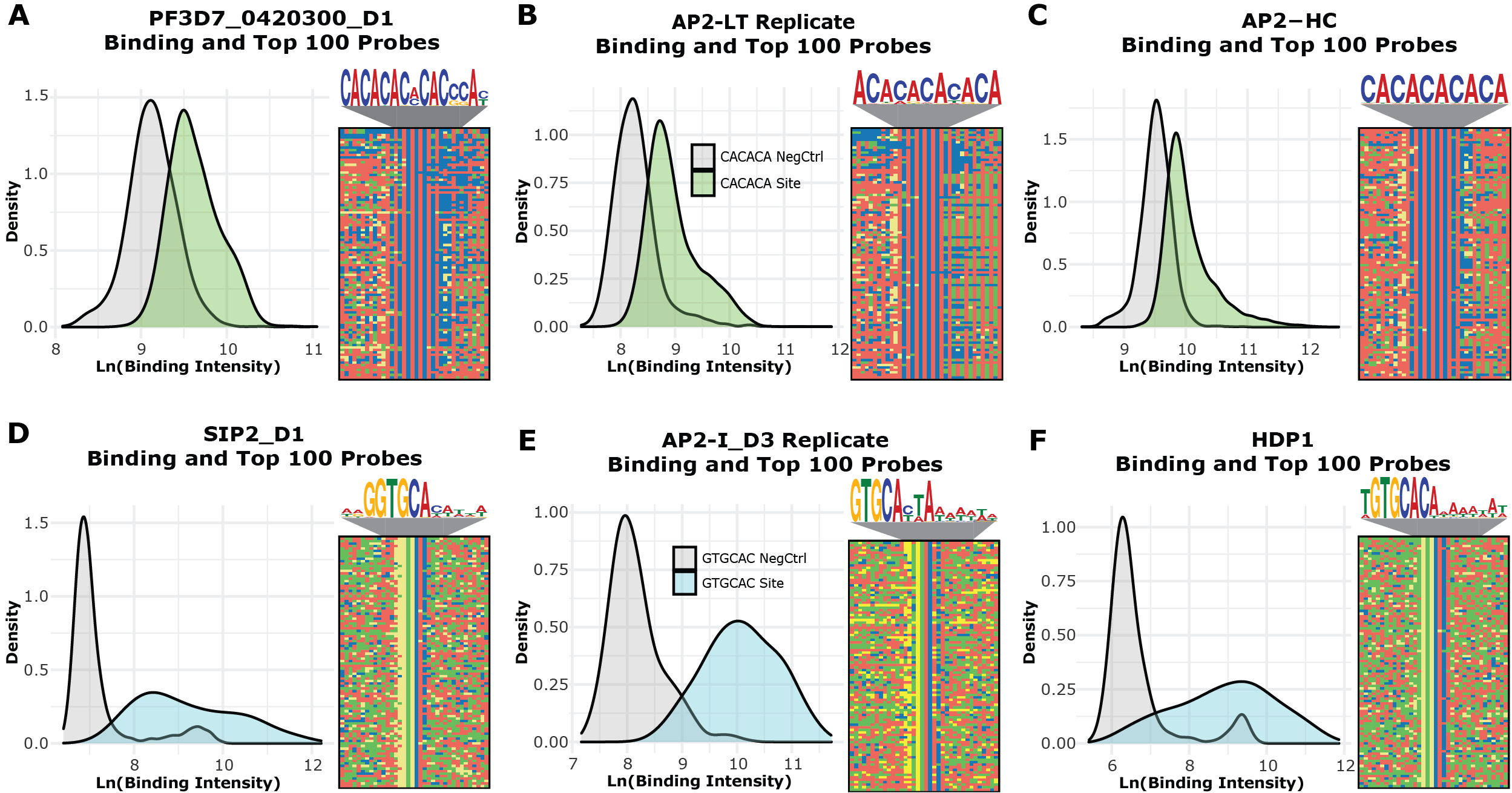


**Supplemental Figure 3: Distribution of binding specificity and top 100 bound probes for DBDs**

**(A-C)** *Left*: Distributions of the binding intensities for the CACACA-binding group including the technical replicate (CACACA probes [Green] and negative control probes [Grey]). *Right*: the DNA motif enriched in the 100 top bound probes with a four-color plot of the 100 top bound probes underneath. Color representations: A (Red), C (Blue), G (Yellow), and T (Green); **(D-F)** *Left*: Distributions of the binding intensities for the GTGCAC-binding group including a technical replicate (GTGCAC probes [Blue] and negative control probes [Grey]).


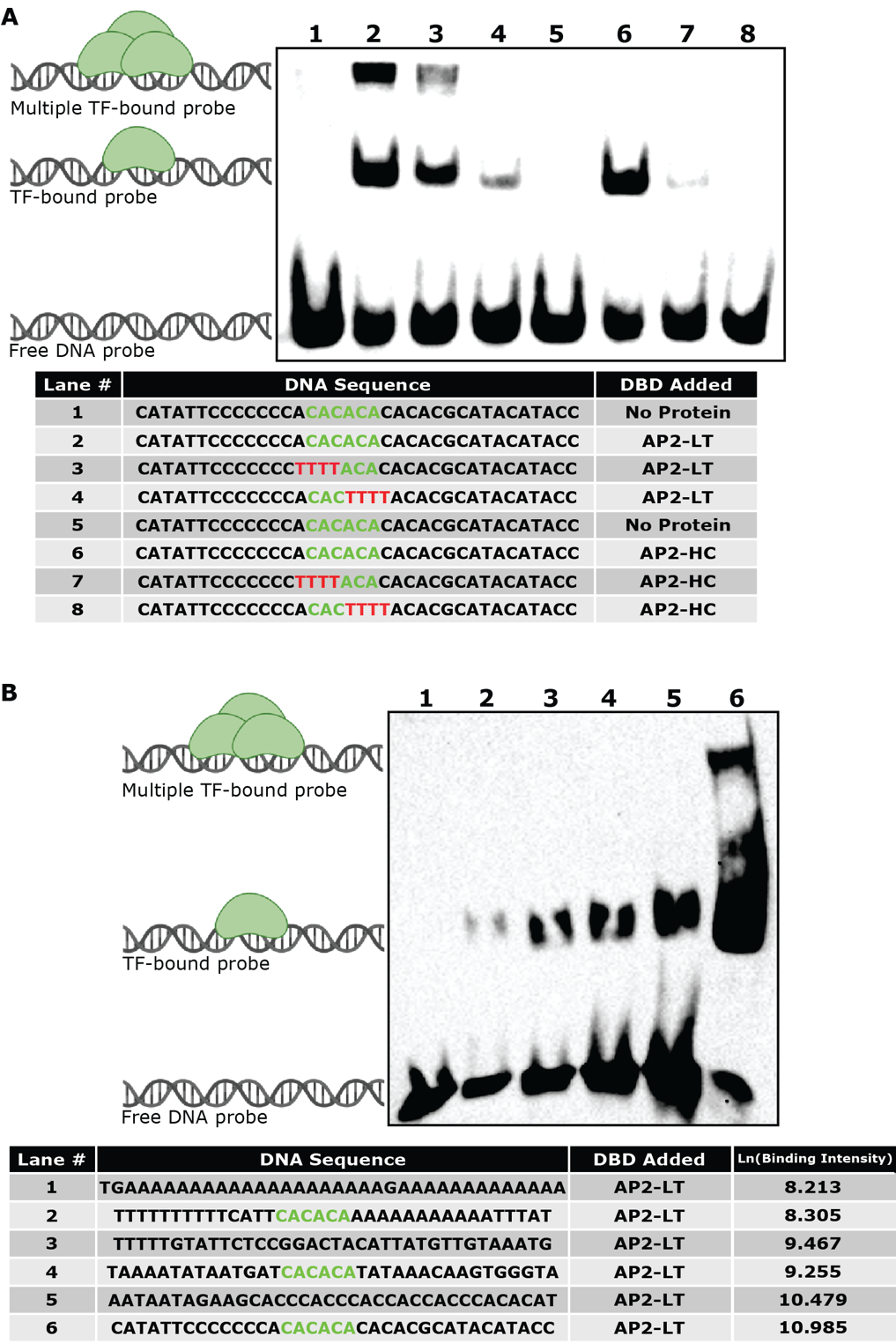


**Supplemental Figure 4: AP2-LT DBD binds to longer CA-dinucleotides with multiple DBDs**

**(A)** Electrophoretic mobility shift assays (EMSAs) with purified AP2-LT and AP2-HC AP2 domains. The DNA sequences associated with the lane numbers are below the gel image (ChemiDoc exposure time 25sec). The protein added to lanes noted below the gel image. The “No protein” notation represents only free probe without protein added as a negative control. Protein-DNA interaction graphic was created with BioRender.com; **(B)** Validation EMSA with purified AP2-LT AP2 domain with different DNA probes that had low-to-high binding in the gcPBM experiments (*from left to right*). (ChemiDoc exposure time 600sec). Protein-DNA interaction graphic was created with BioRender.com.


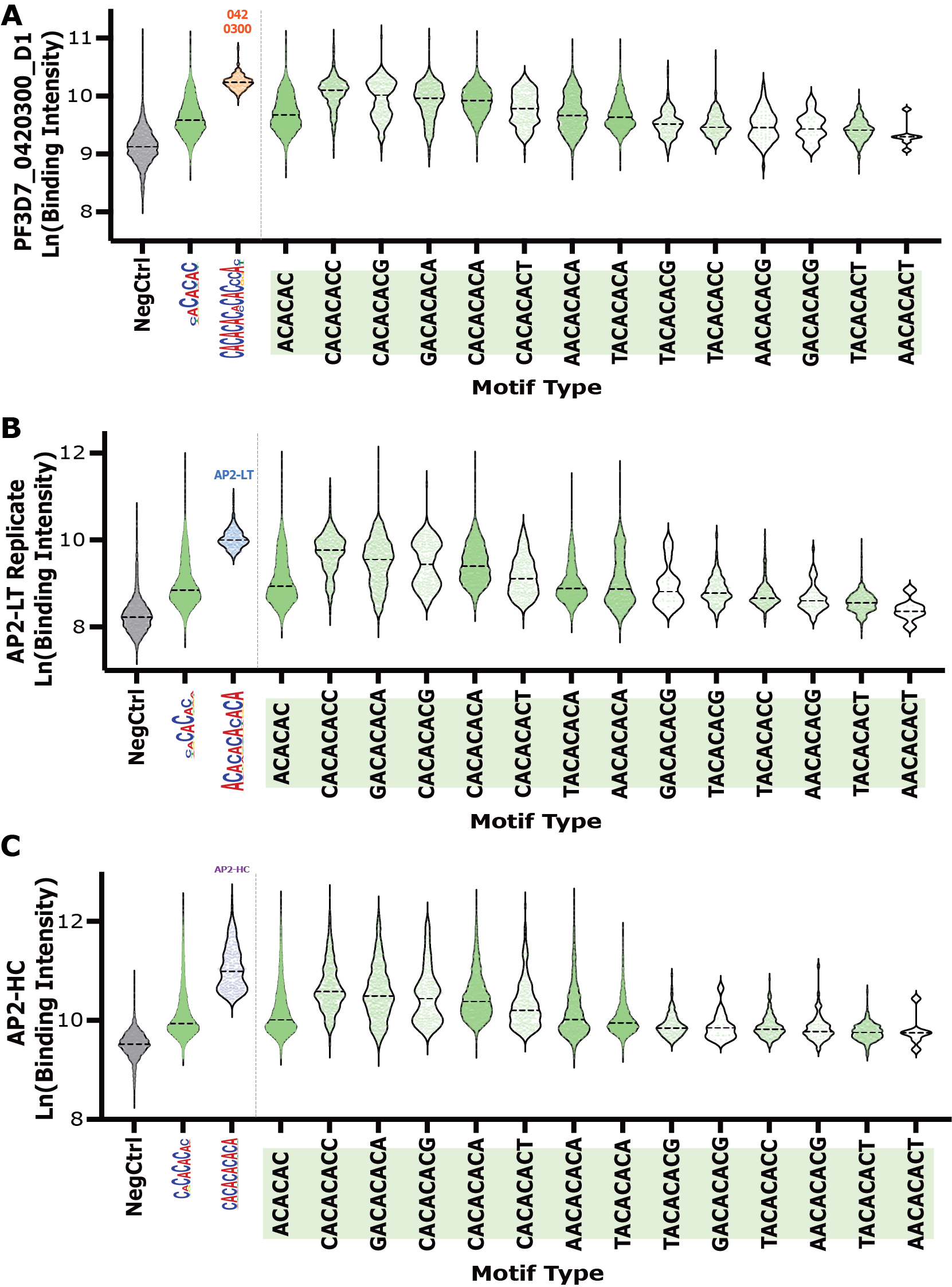


**Supplemental Figure 5: CACACA-binding DBDs have minimal differences in sequence context preferences**

**(A)** Binding intensity distributions for CACACA negative control probes (Grey), all CACACA probes (Green), the PF3D7_0420300_D1 extended motif probes (Orange), and 8-mer CACACA probes represented in the gcPBM (Green). Dotted lines are the calculated mean for each violin plot; **(B)** Binding intensity distributions for CACACA negative control probes (Grey), all CACACA probes (Green), the AP2-LT Replicate extended motif probes (Blue), and 8-mer CACACA probes represented in the gcPBM (Green). Dotted lines are the calculated mean for each violin plot; **(C)** Binding intensity distributions for CACACA negative control probes (Grey), all CACACA probes (Green), the AP2-HC extended motif probes (Purple), and 8-mer CACACA probes represented in the gcPBM (Green). Dotted lines are the calculated mean for each violin plot.


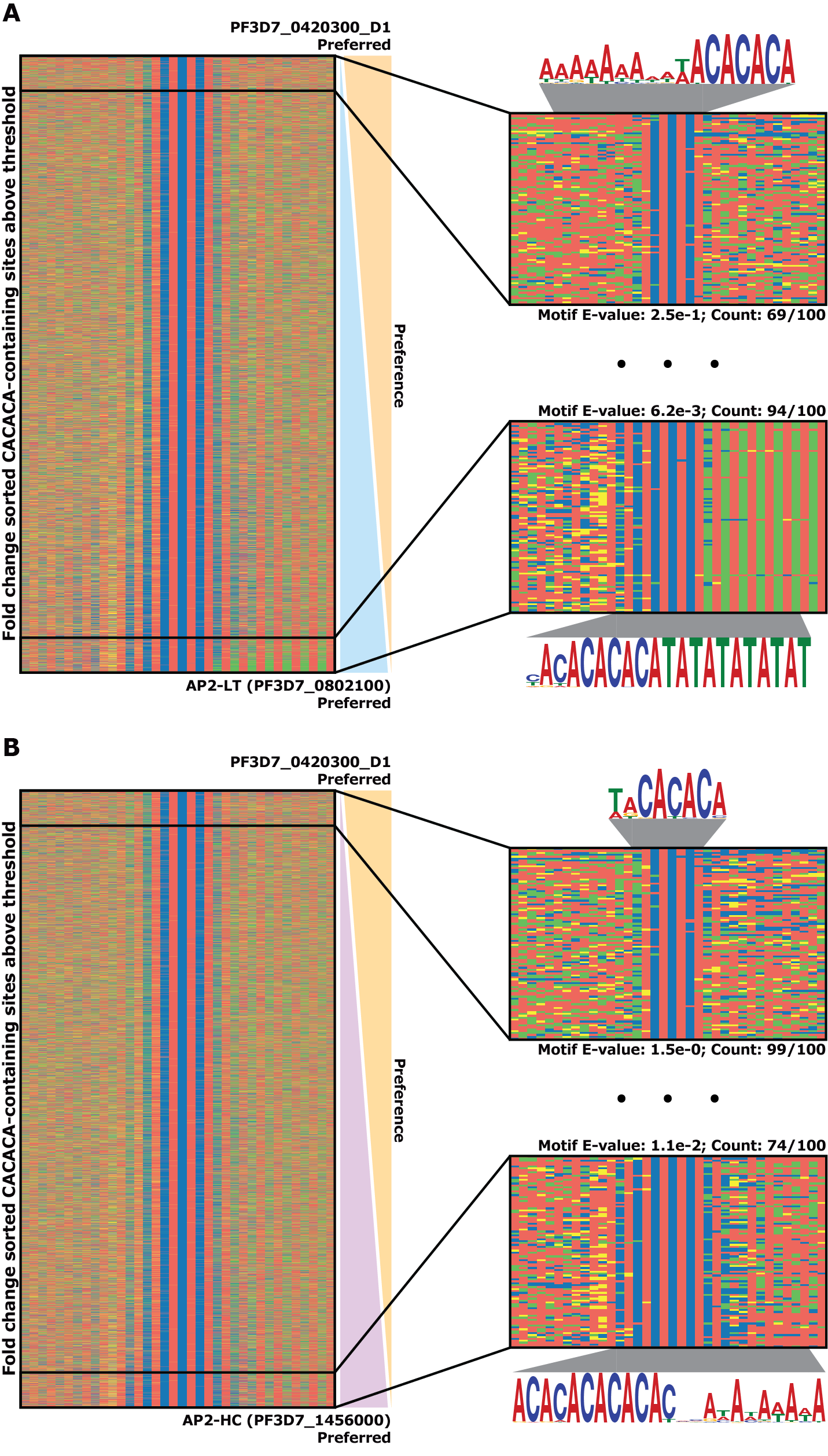


**Supplemental Figure 6: Differential sequence preferences of CACACA-binding DBDs**

***(A****) Left:* Four-color plot of CACACA probes above the 90^th^ percentile of negative control probes sorted by fold change (log_2_[PF3D7_0420300/AP2-LT]). *Right:* Zoom in on the top 100 differentially bound probes by PF3D7_0420300_D1 (*top right*) and AP2-LT (*bottom right*) with enriched motifs, calculated E-values, and motif occurrence counts within the top 100 sites. Color representations: A (Red), C (Blue), G (Yellow), and T (Green); **(B)** *Left:* Four-color plot of CACACA probes above the 90^th^ percentile of negative control probes sorted by fold change (log_2_[PF3D7_0420300/AP2-HC]). *Right:* Zoom in on the top 100 differentially bound probes by PF3D7_0420300_D1 (*top right*) and AP2-HC (*bottom right*) with enriched motifs, calculated E-values, and motif occurrence counts within the top 100 sites.


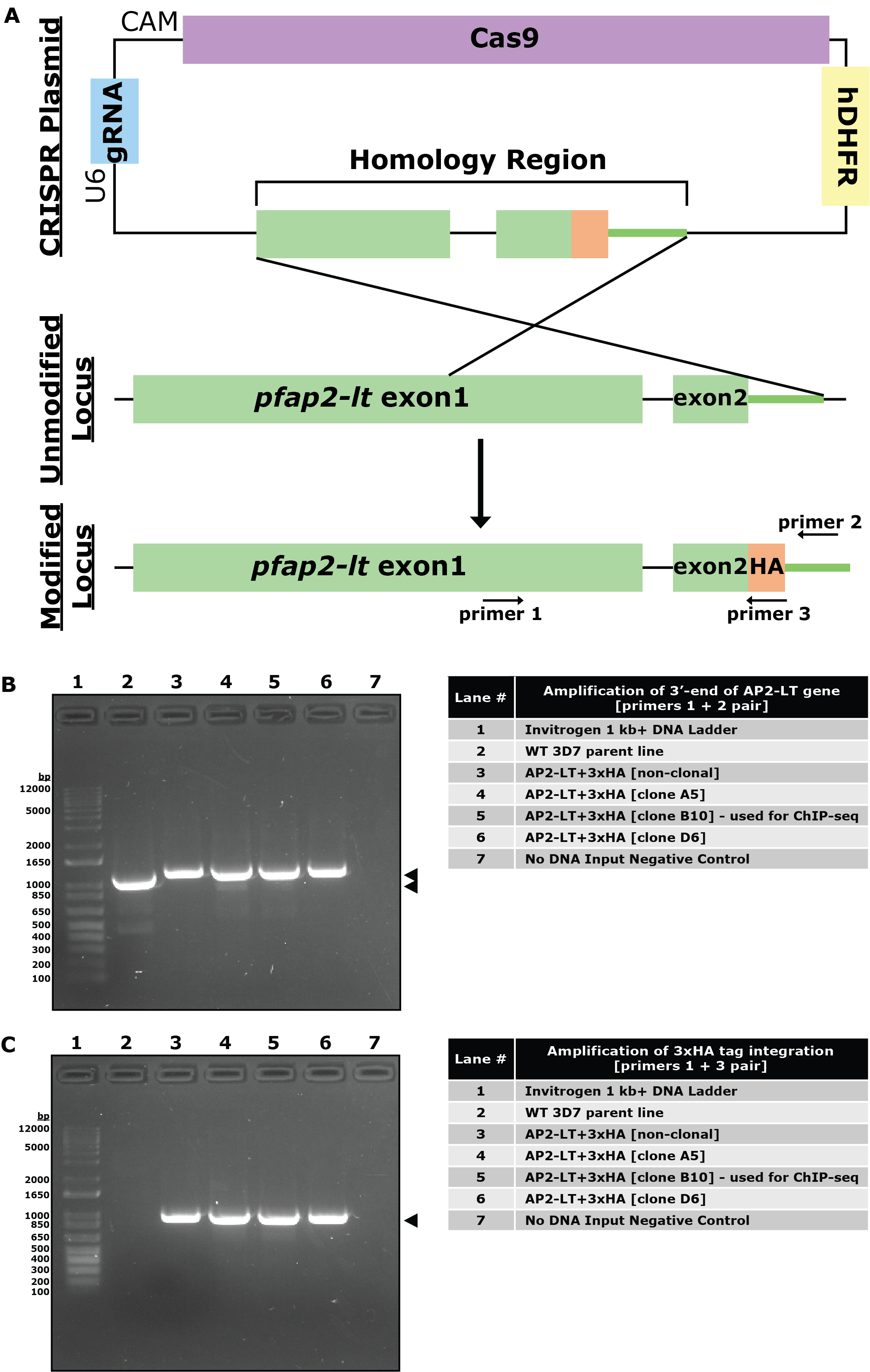


**Supplemental Figure 7: Generation of PfAP2-LT^HA^ tagged line for ChIP-seq experiments**

**(A)** Schematic of endogenous gene locus tagging by double homologous recombination via CRISPR-Cas9; **(B)** Genotyping PCR to amplify the 3`-end of *pfap2-lt* with primers 1 and 2 (notated in Panel A); **(C)** Genotyping PCR to amplify only if the tag was integrated into the endogenous *pfap2-lt* locus with primers 1 and 3 (notated in Panel A).


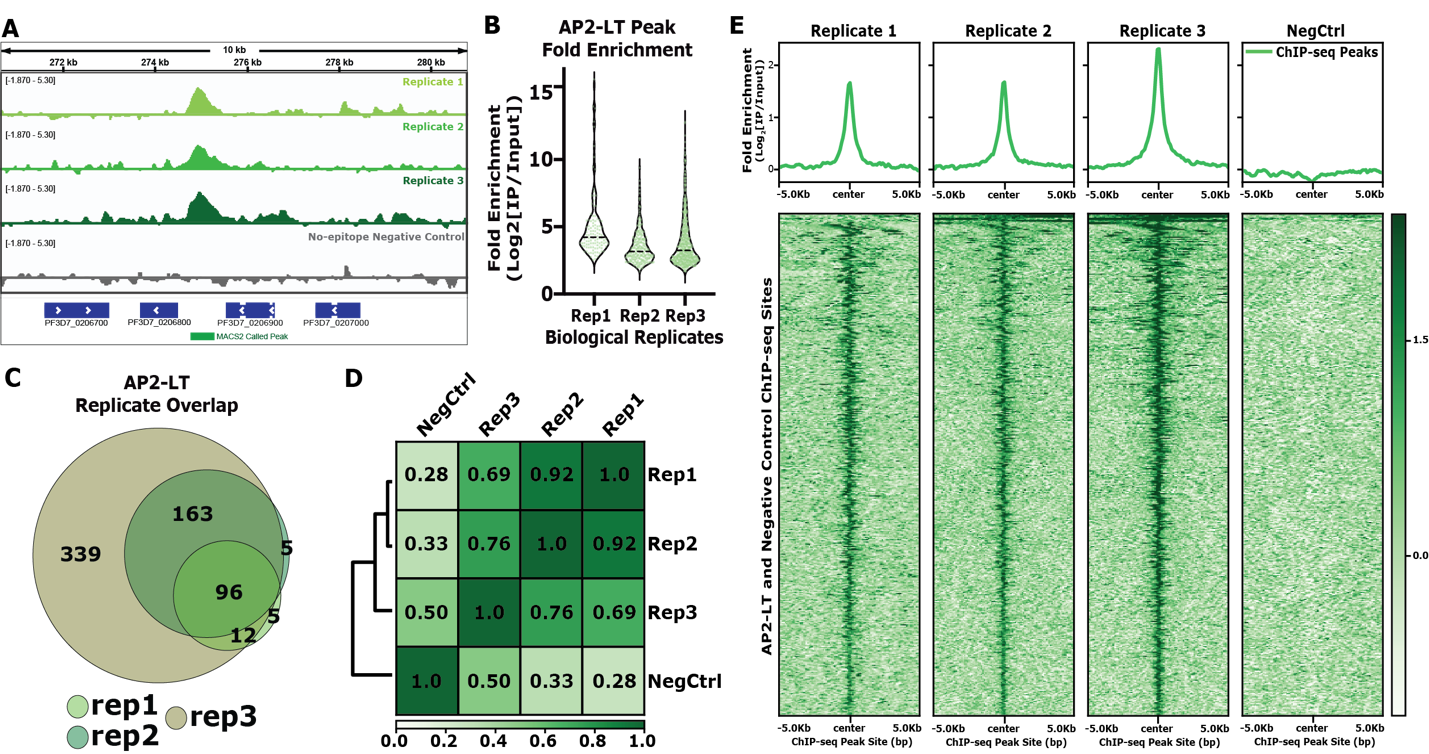


**Supplemental Figure 8: Quality control for AP2-LT ChIP-seq experiments**

(**A**) Example screenshot from Integrative Genomics Viewer (IGV) of a 10kb region on chromosome 2. ChIP-seq genome tracks depicted from top to bottom are as follows: AP2-LT biological replicate one (log_2_[IP/Input]) [Light Green], AP2-LT biological replicate two (log_2_[IP/Input]) [Medium Green], AP2-LT biological replicate three (log_2_[IP/Input]) [Dark Green], No-epitope Negative Control single replicate (log_2_[IP/Input]) [Dark Grey], *P. falciparum* 3D7 strain gene annotation (Pfalciparum3D7, version 3, release 38) [Dark Blue], and significantly (q-value < 0.01) called peak region by MACS2 [Green bar]; (**B**) Distribution of fold enrichment (Log_2_[IP/Input]) of AP2-LT ChIP-seq MACS2-called peaks from each replicate experiment; (**C**) MACS2-called peak ChIP-seq replicate overlaps; (**D**) Pearson correlation plot of AP2-LT ChIP-seq three biological replicates and no-epitope negative control ChIP-seq sample; (**E**) *Top:* Profile plot of the mean AP2-LT ChIP-seq fold enrichment (Log_2_[IP/Input]) across all three biological replicates and no-epitope negative control sample. *Bottom:* Heatmap of the AP2-LT ChIP-seq fold enrichment (Log_2_[IP/Input]) across all three biological replicates and no-epitope negative control sample.


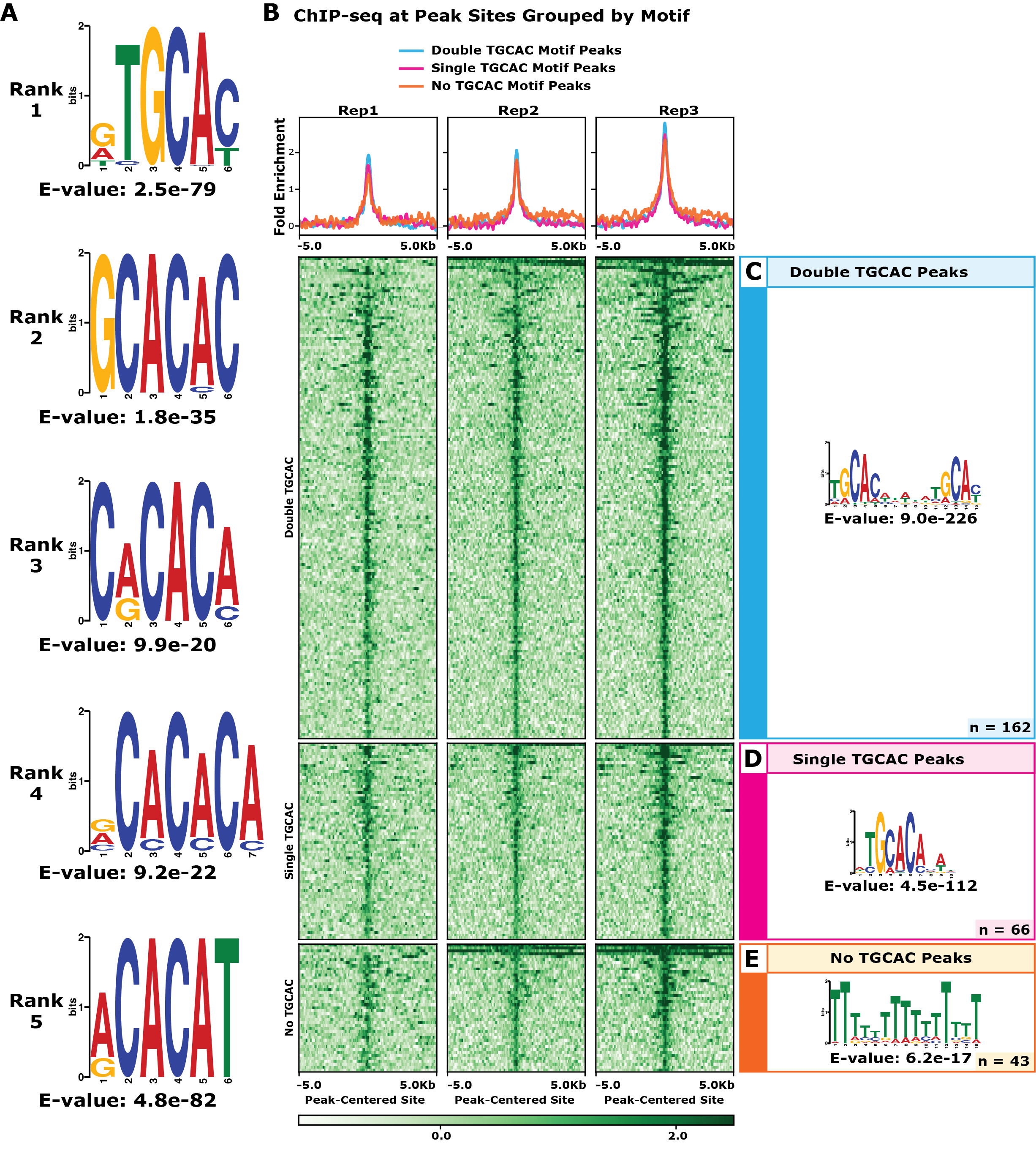


**Supplemental Figure 9: Motif enrichment analyses for single and double TGCAC motif in AP2-LT ChIP-seq binding sites**

(**A**) Top 5 ranked (non-A/T rich) DNA sequence motifs differentially enriched between ChIP-seq peaks and background regions with a minimum motif length of 6 and a maximum length of 25; **(B)** *Top:*Profile plot of the mean AP2-LT ChIP-seq fold enrichment (Log_2_[IP/Input]) across all three biological replicates and categorized into three different regions (Double TGCAC motif peaks [Blue], Single TGCAC motif peaks [Pink], and no TGCAC motif peaks [Orange]).*Bottom:*Heatmap of the AP2-LT ChIP-seq fold enrichment (Log_2_[IP/Input]) across all three biological replicates and categorized into three different non-overlapping regions (shown above); **(C)**Motif enrichment and E-value for sites with the double TGCAC motif; **(D)** Motif enrichment and E-value for single TGCAC motif peaks that did not contain the double motif; and **(E)** Motif enrichment and E-value for No TGCAC motif peaks.


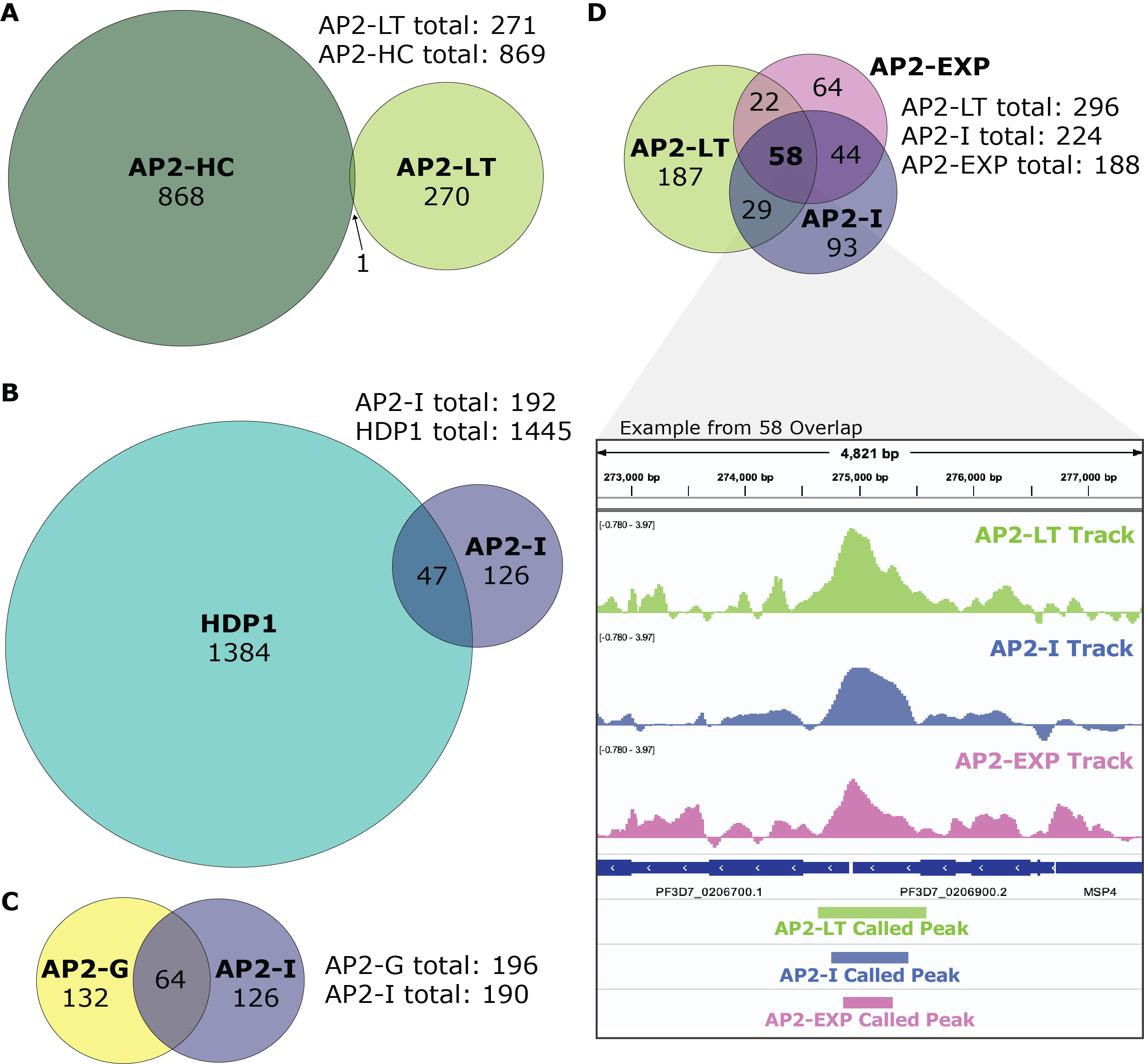


**Supplemental Figure 10: Comparisons of *in vivo* genome-wide occupancies of *P. falciparum* TFs in this study**

**(A)** Comparison of the genomic regions bound by AP2-LT (this study) and published AP2-HC; (**B**) Comparison of the genomic regions bound by HDP1 and AP2-I from published ChIP-seq experiments; (**C**) Comparison of the genomic regions bound by published AP2-G and AP2-I; (**D**) Comparison of the genomic regions bound by AP2-LT (this study), published AP2-I, and published AP2-EXP. *Bottom*: screenshot from Integrative Genomics Viewer (IGV) of a representative overlapping locus with AP2-LT (Green), AP2-I (Blue), and AP2-EXP (Pink). Significantly bound regions identified by MACS2 peak caller represented as genomic interval bars under peak data.


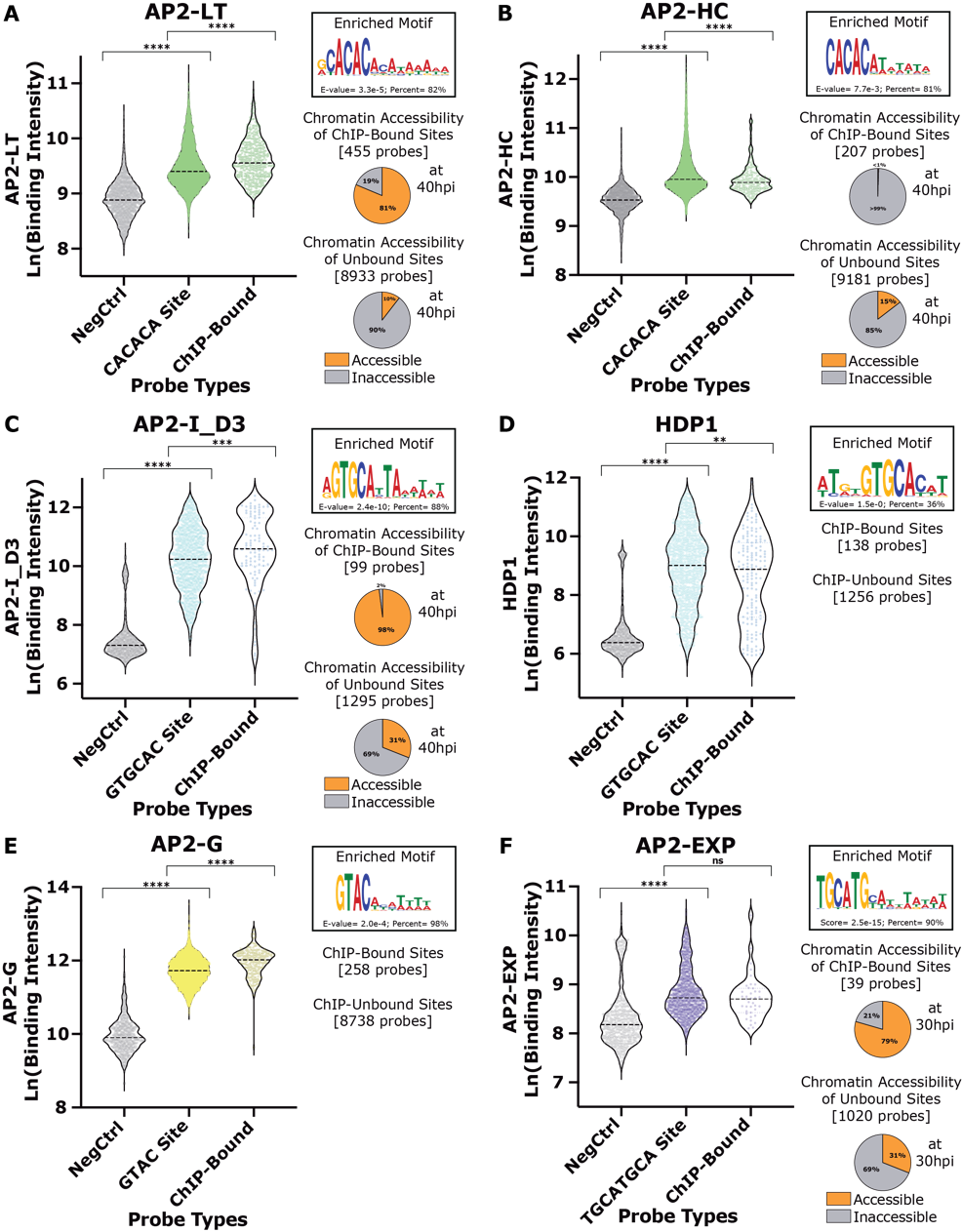


**Supplemental Figure 11: Motif enrichment and chromatin accessibility of ChIP-bound and ChIP-unbound sites for six TFs**

(**A**) *Left:* Binding intensity distributions for negative control sites (Grey), all CACACA motif sites (Green), and CACACA-containing sites that are AP2-LT ChIP-bound (Green). *Right Top:* AP2-LT ChIP-bound enriched motif, calculated E-value, and percent of occurrences. *Right Bottom:* Pie charts of chromatin accessibility and inaccessibility for ChIP-bound (top) and ChIP-unbound (bottom). Two-tailed Mann-Whitney test [p-value < 0.0001(****), 0.001(***), 0.01(**), or not significant(ns)]; (**B**) Binding intensity distributions, enriched motif and chromatin accessibility with AP2-HC data; (**C**) Binding intensity distributions, enriched motif and chromatin accessibility with AP2-I data; (**D**) Binding intensity distributions and enriched motif with HDP1 data. Chromatin accessibility data is not available for the life cycle stage HDP1 ChIP-seq was taken (stage II gametocytes); (**E**) Binding intensity distributions and enriched motif with AP2-G data. Chromatin accessibility data is not available for the life cycle stage AP2-G ChIP-seq was taken (committing gametocytes); (**F**) Binding intensity distributions, enriched motif and chromatin accessibility with AP2-EXP data.


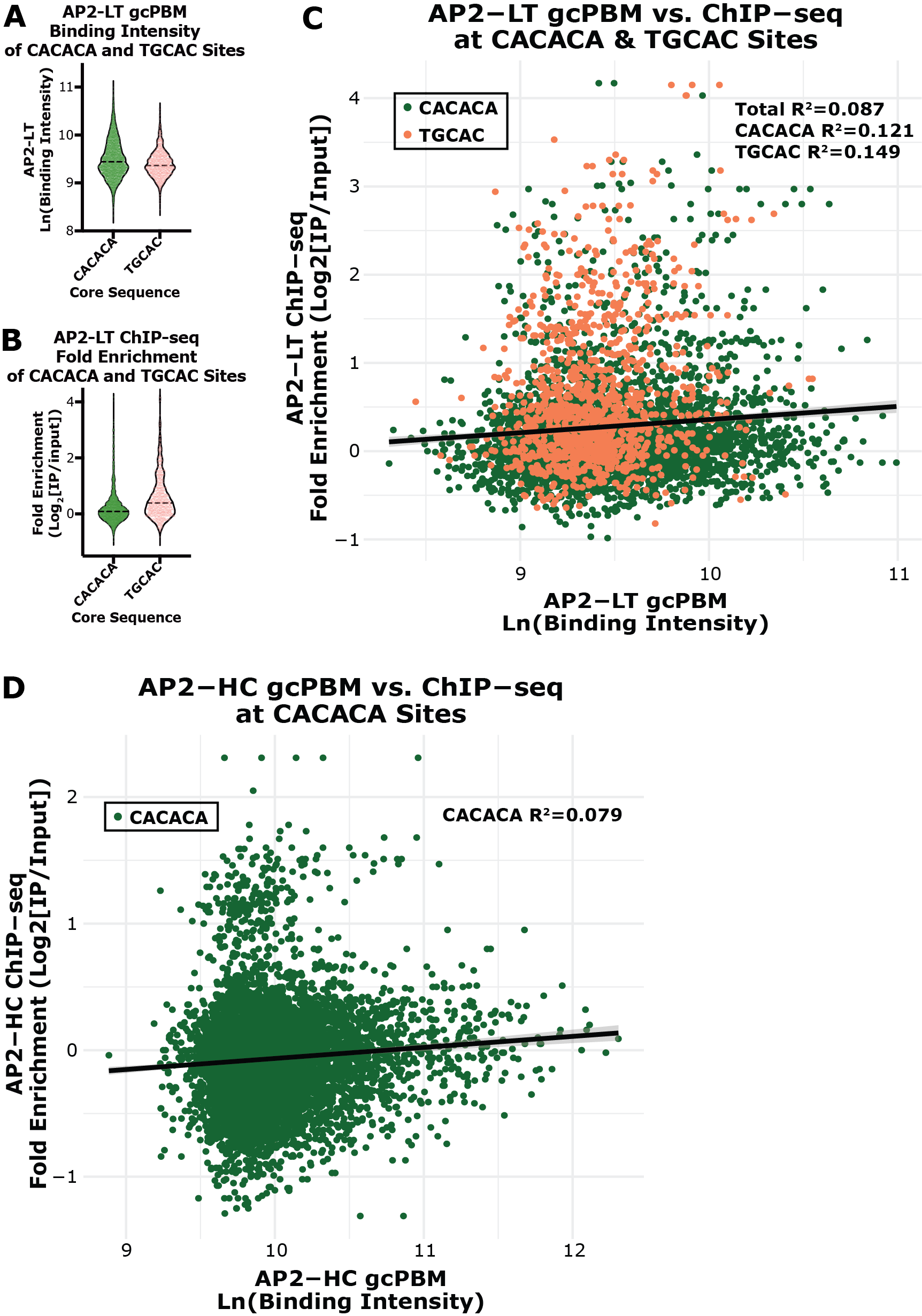


**Supplemental Figure 12: Comparing AP2-LT and AP2-HC binding *in vitro* and *in vivo***

(**A**) AP2-LT gcPBM binding intensity across CACACA sites (Green) and TGCAC (Peach); (**B**) AP2-LT ChIP-seq fold enrichment (Log_2_[IP/Input]) across CACACA sites (Green) and TGCAC (Peach); (**C**) Comparison of AP2-LT gcPBM binding intensity and AP2-LT ChIP-seq fold enrichment (Log_2_[IP/Input]) across CACACA sites (Green) and TGCAC (Peach). *Top Right:* Pearson correlation values for all data points, only CACACA data points, and TGCAC data points. (**D**) Comparison of AP2-HC gcPBM binding intensity and AP2-HC ChIP-seq fold enrichment (Log_2_[IP/Input]) across CACACA sites (Green). *Top Right:* Pearson correlation values for all data points.


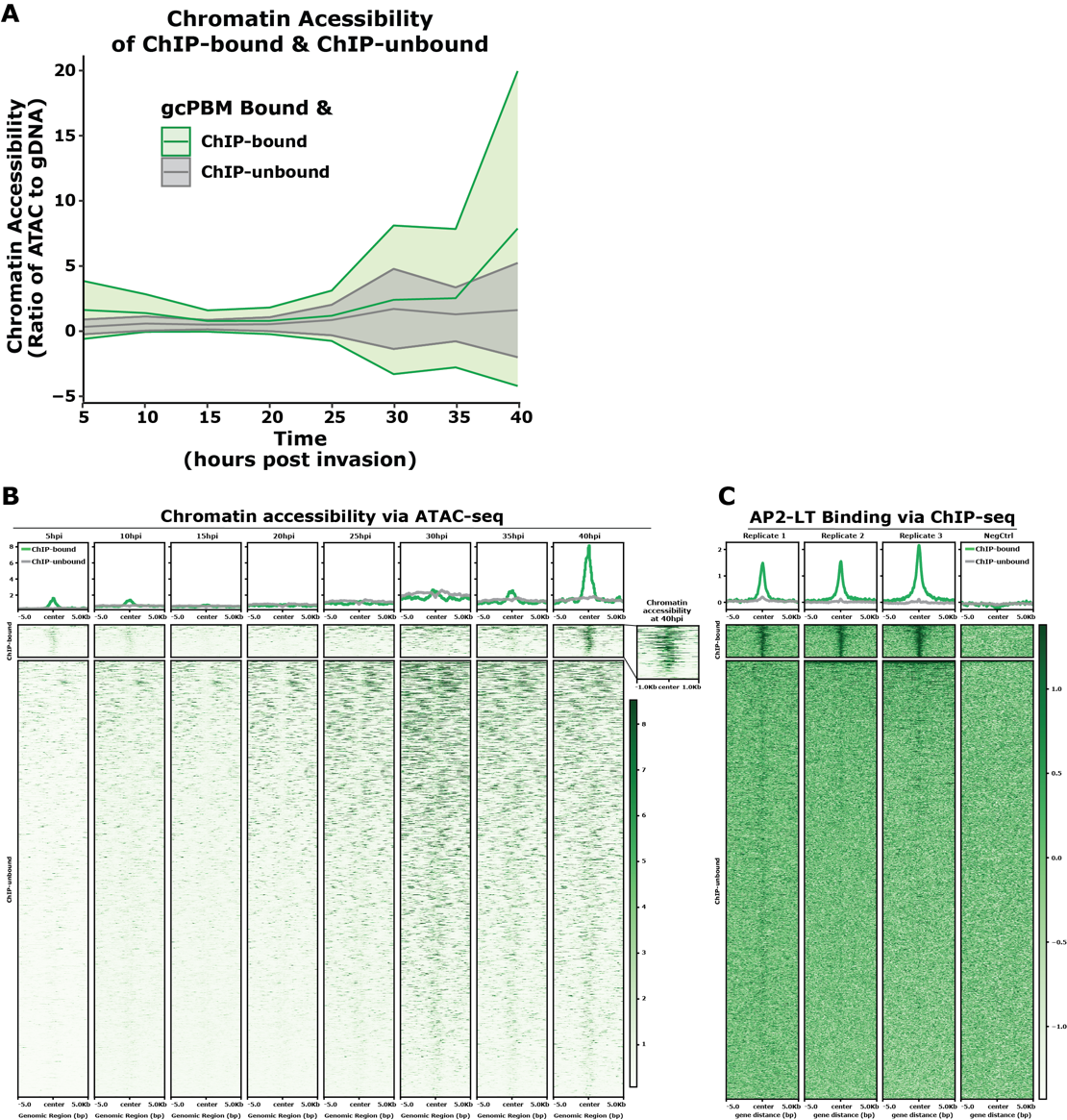


**Supplemental Figure 13: Chromatin accessibility of AP2-LT ChIP-bound and ChIP-unbound CACACA-containing sites**

(**A**) Line plot of chromatin accessibility across eight asexual stage timepoints (5hpi, 10hpi, 15hpi, 20hpi, 25hpi, 30hpi, 35hpi, and 40hpi) for AP2-LT ChIP-bound (Green) and ChIP-unbound (Grey) sites. Central line plotted is the median normalized read count over gDNA control. Upper and lower lines are the median plus and minus one standard deviation; (**B**) *Top:* Profile plot of the mean chromatin accessibility (median normalized read count over gDNA control) across eight asexual stage timepoints (5hpi, 10hpi, 15hpi, 20hpi, 25hpi, 30hpi, 35hpi, and 40hpi) for AP2-LT ChIP-bound (Green) and ChIP-unbound (Grey) sites. *Bottom:* Heatmap of chromatin accessibility (median normalized read count over gDNA control) across eight asexual stage timepoints (5hpi, 10hpi, 15hpi, 20hpi, 25hpi, 30hpi, 35hpi, and 40hpi) for AP2-LT ChIP-bound (top) and ChIP-unbound (bottom) sites. *Top Right zoom in:* Chromatin accessibility of AP2-LT ChIP-bound CACACA-containing sites at 40hpi; and (**C**) *Top:* Profile plot of the mean AP2-LT ChIP-seq fold enrichment (Log_2_[IP/Input]) for all three biological replicates and no-epitope negative control sample across AP2-LT ChIP-bound (Green) and ChIP-unbound (Grey) sites. *Bottom:* Heatmap of the AP2-LT ChIP-seq fold enrichment (Log_2_[IP/Input]) for all three biological replicates and no-epitope negative control sample AP2-LT ChIP-bound (Green) and ChIP-unbound (Grey) sites.


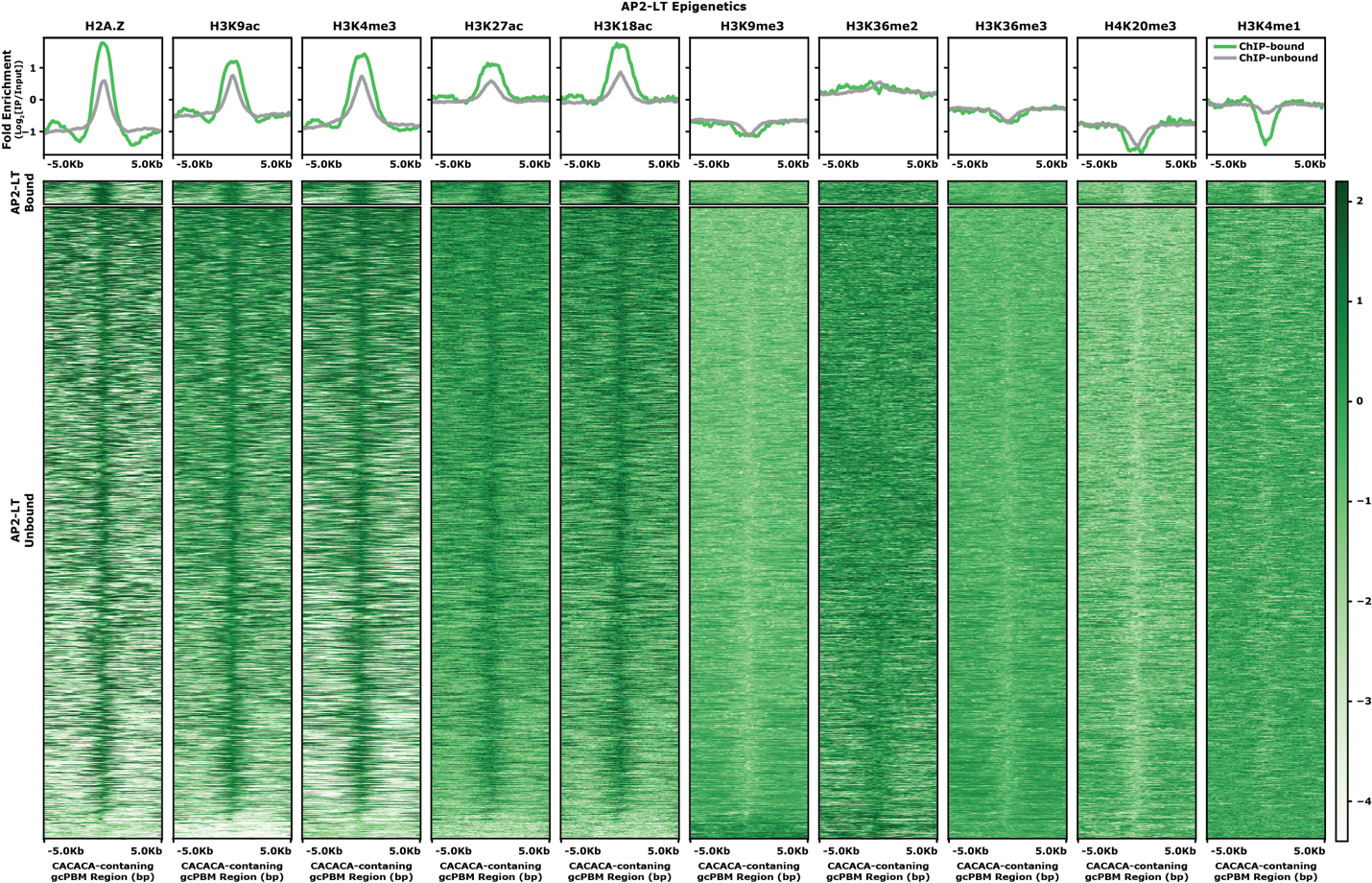


**Supplemental Figure 14: Epigenetic landscape of AP2-LT ChIP-bound and ChIP-unbound CACACA-containing sites**

*Top:* Profile plot of the mean ChIP-seq fold enrichment (Log_2_[IP/Input]) of five activation epigenetic marks (H2A.Z, H3K9ac, H3K4me3, H3K27ac, and H3K18ac) and five repression epigenetic marks (H3K9me3, H3K36me2/3, H4K20me3, and H3K4me1) for AP2-LT ChIP-bound (Green) and ChIP-unbound (Grey) sites. *Bottom:* Heatmap of ChIP-seq fold enrichment (Log_2_[IP/Input]) of five activation epigenetic marks (H2A.Z, H3K9ac, H3K4me3, H3K27ac, and H3K18ac) and five repression epigenetic marks (H3K9me3, H3K36me2/3, H4K20me3, and H3K4me1) for AP2-LT ChIP-bound (Green) and ChIP-unbound (Grey) sites.


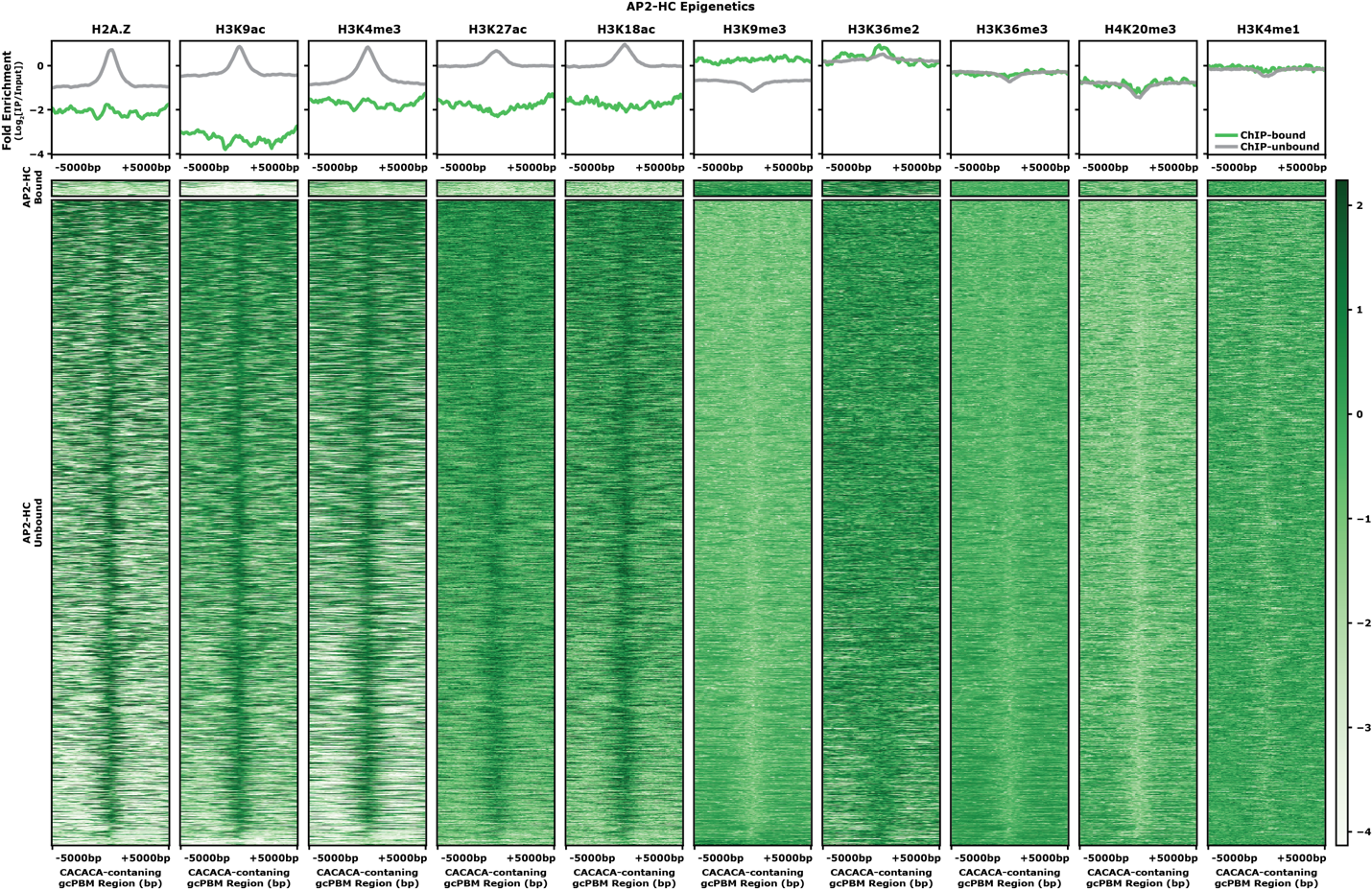


**Supplemental Figure 15: Epigenetic landscape of AP2-HC ChIP-bound and ChIP-unbound CACACA-containing sites**

*Top:* Profile plot of the mean ChIP-seq fold enrichment (Log_2_[IP/Input]) of five activation epigenetic marks (H2A.Z, H3K9ac, H3K4me3, H3K27ac, and H3K18ac) and five repression epigenetic marks (H3K9me3, H3K36me2/3, H4K20me3, and H3K4me1) for AP2-HC ChIP-bound (Green) and ChIP-unbound (Grey) sites. *Bottom:* Heatmap of ChIP-seq fold enrichment (Log_2_[IP/Input]) of five activation epigenetic marks (H2A.Z, H3K9ac, H3K4me3, H3K27ac, and H3K18ac) and five repression epigenetic marks (H3K9me3, H3K36me2/3, H4K20me3, and H3K4me1) for AP2-HC ChIP-bound (Green) and ChIP-unbound (Grey) sites.


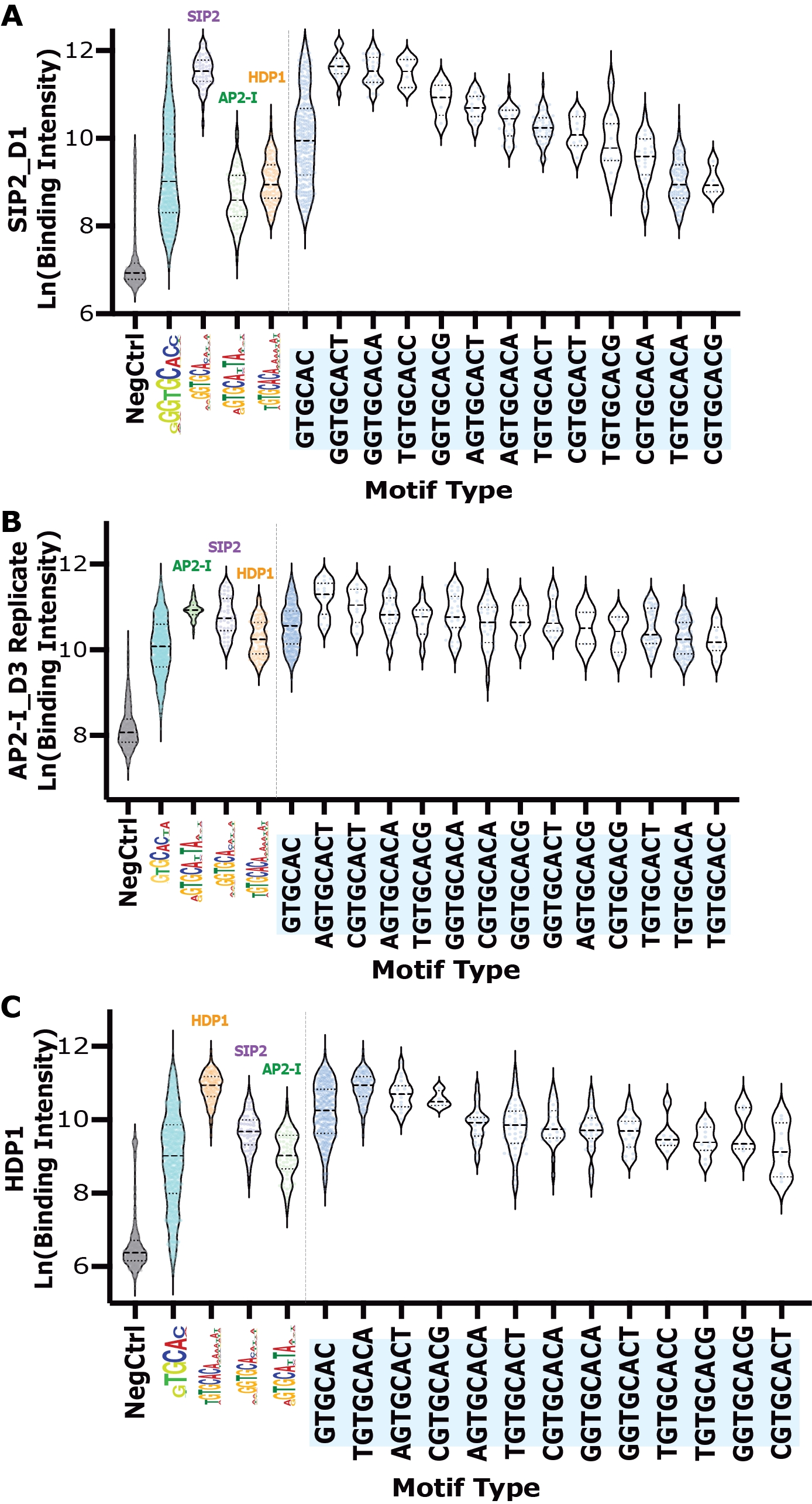


**Supplemental Figure 16: GTGCAC-binding DBDs have divergent sequence context preferences**

**(A)** Binding intensity distributions of SIP2_D1 for GTGCAC negative control probes (Grey), all GTGCAC probes (Blue), the extended motif probes by all three GTGCAC-binding TFs (AP2-I_D3[Green], SIP2_D1[Purple], and HDP1[Orange]), and 8-mer GTGCAC probes represented in the gcPBM (Blue). Dotted lines are the calculated mean for each violin plot; **(B)** Binding intensity distributions of AP2-I_D3 Replicate for GTGCAC negative control probes (Grey), all GTGCAC probes (Blue), the extended motif probes by all three GTGCAC-binding TFs (AP2-I_D3[Green], SIP2_D1[Purple], and HDP1[Orange]), and 8-mer GTGCAC probes represented in the gcPBM (Blue). Dotted lines are the calculated mean for each violin plot; **(C)** Binding intensity distributions of HDP1 for GTGCAC negative control probes (Grey), all GTGCAC probes (Blue), the extended motif probes by all three GTGCAC-binding TFs (AP2-I_D3[Green], SIP2_D1[Purple], and HDP1[Orange]), and 8-mer GTGCAC probes represented in the gcPBM (Blue). Dotted lines are the calculated mean for each violin plot.


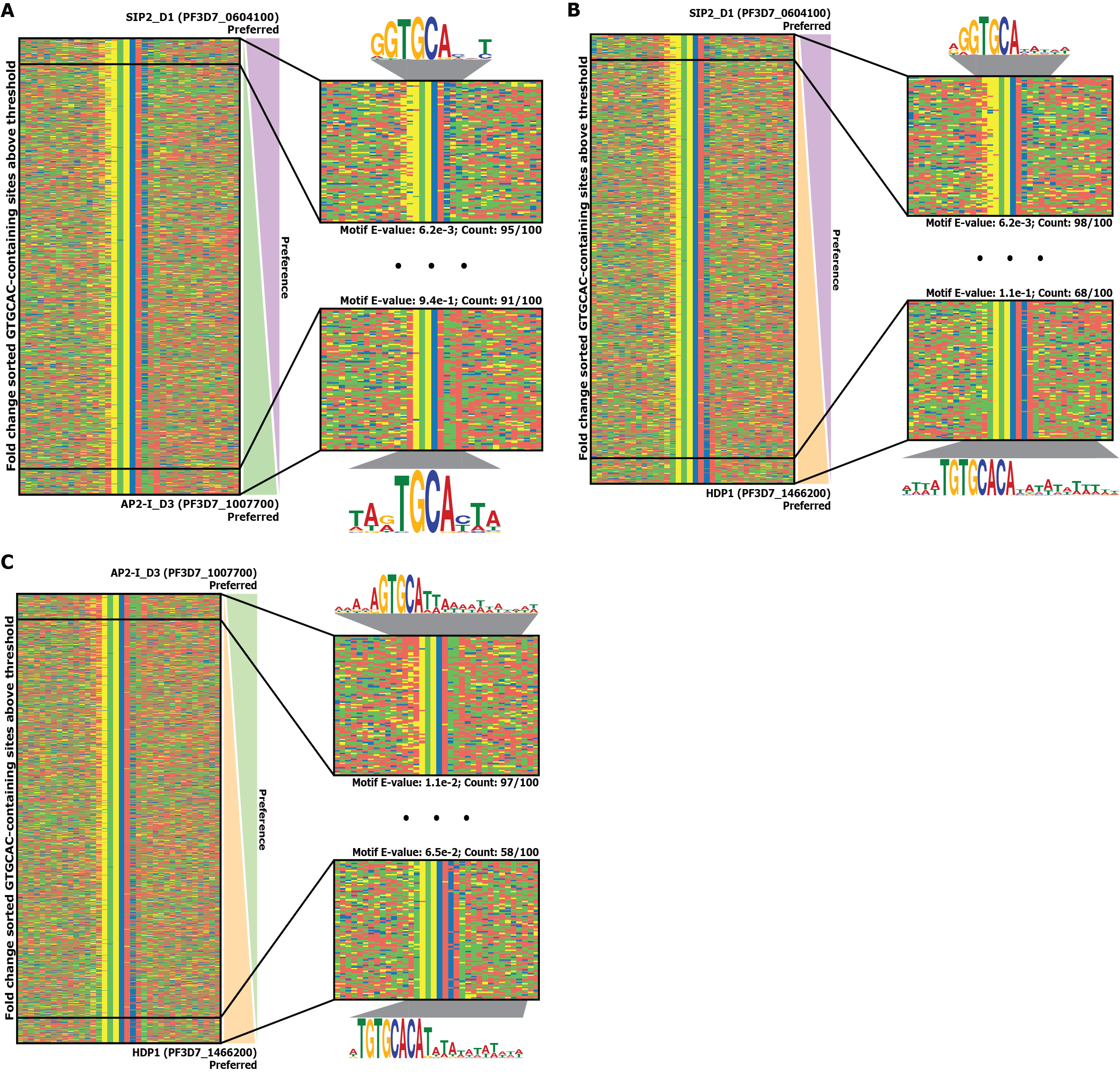


**Supplemental Figure 17: Differential sequence preference of GTGCAC-binding TFs**

**(A)** *Left*: Four-color plot of all GTGCAC probes in gcPBM design above 90^th^ percentile of the negative control probes, sorted by fold change (Log_2_[SIP2/AP2-I]). *Right*: Enriched DNA motif (by Meme Suite: Streme) and zoom in of the top 100 bound probes by each SIP2_D1 (*top*) and AP2-I_D3 (*bottom*). Color representations: A (Red), C (Blue), G (Yellow), and T (Green); (**B**) *Left*: Four-color plot of all GTGCAC probes in gcPBM design above 90^th^ percentile of the negative control probes, sorted by fold change (Log_2_[SIP2/HDP1]). *Right*: Enriched DNA motif (by Meme Suite: Streme) and zoom in of the top 100 bound probes by each SIP2_D1 (*top*) and HDP1 (*bottom*); (**C**) *Left*: Four-color plot of all GTGCAC probes in gcPBM design sorted by fold change (Log_2_[AP2-I/HDP1]). *Right*: Enriched DNA motif (by Meme Suite: Streme) and zoom in of the top 100 bound probes by each AP2-I_D3 (*top*) and HDP1 (*bottom*).


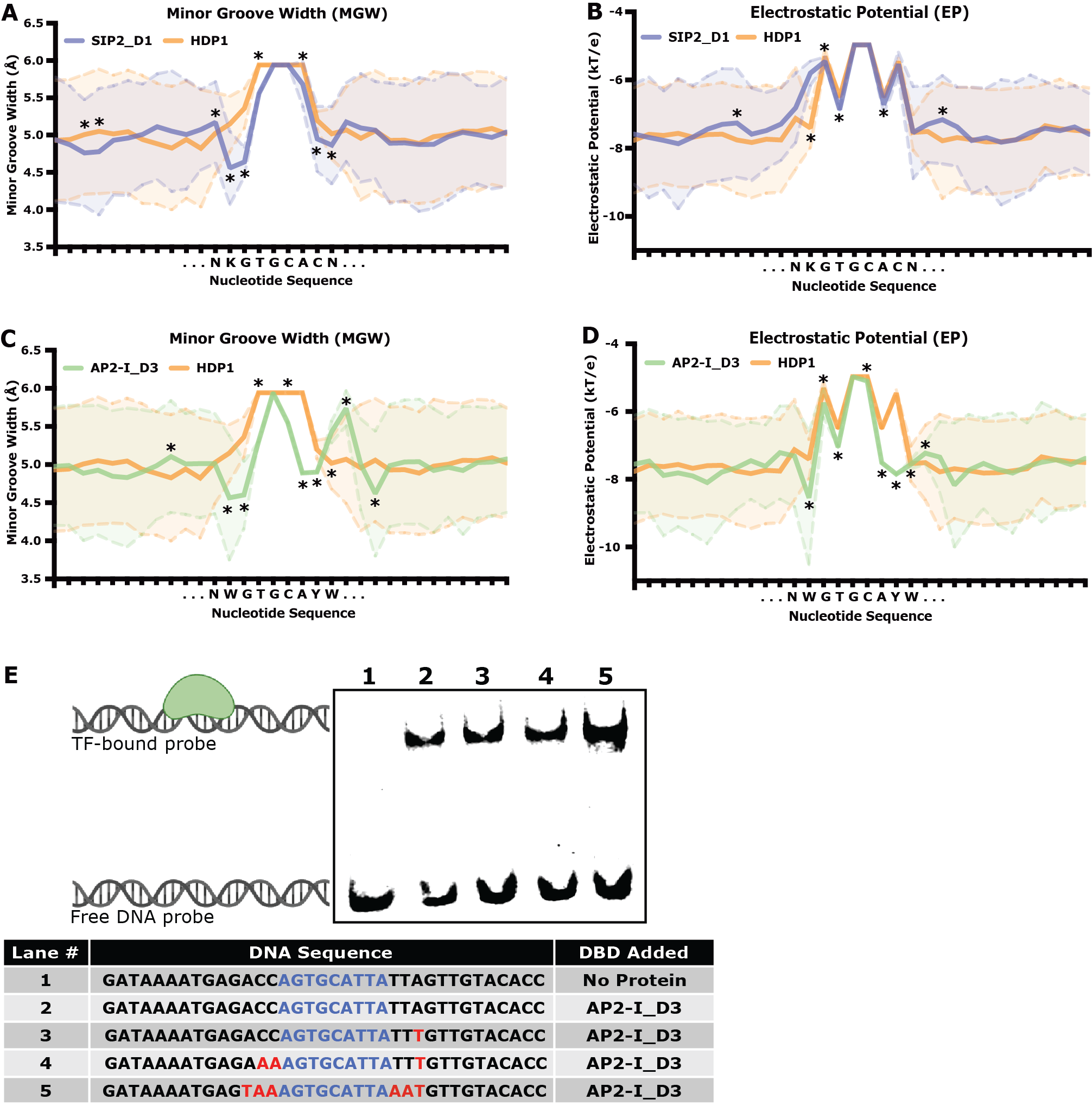


**Supplemental Figure 18: Differential DNA shape preference of GTGCAC-binding TFs**

**(A-B)** Calculated minor groove width (MGW) and electrostatic potential (EP) profiles (using DNAShapeR) for SIP2_D1-preferred-GGTGCAC probes (Purple) and HDP1-preferred-TGTGCACA (Orange) probes. Significant differences (p-value < 0.05) between features notated with an * (two-sided Wilcoxon rank sum test). N = IUPAC for any nucleotide. K = IUPAC for G or T nucleotides; **(C-D)** Calculated MGW and EP profiles (using DNAShapeR) for AP2-I_D3-preferred-AGTGCATTA (Green) probes and HDP1-preferred-TGTGCACA (Orange) probes. Significant differences (p-value < 0.05) between features notated with an * (two-sided Wilcoxon rank sum test). N = IUPAC for any nucleotide. W = IUPAC for A or T nucleotides. Y = IUPAC for C or T nucleotides; (**E**) EMSA of AP2-I_D3 binding to a AGTGCATTA probes with increasing numbers of shape mutants to the sequences outside the extended motif. Protein-DNA interaction graphic was created with BioRender.com.


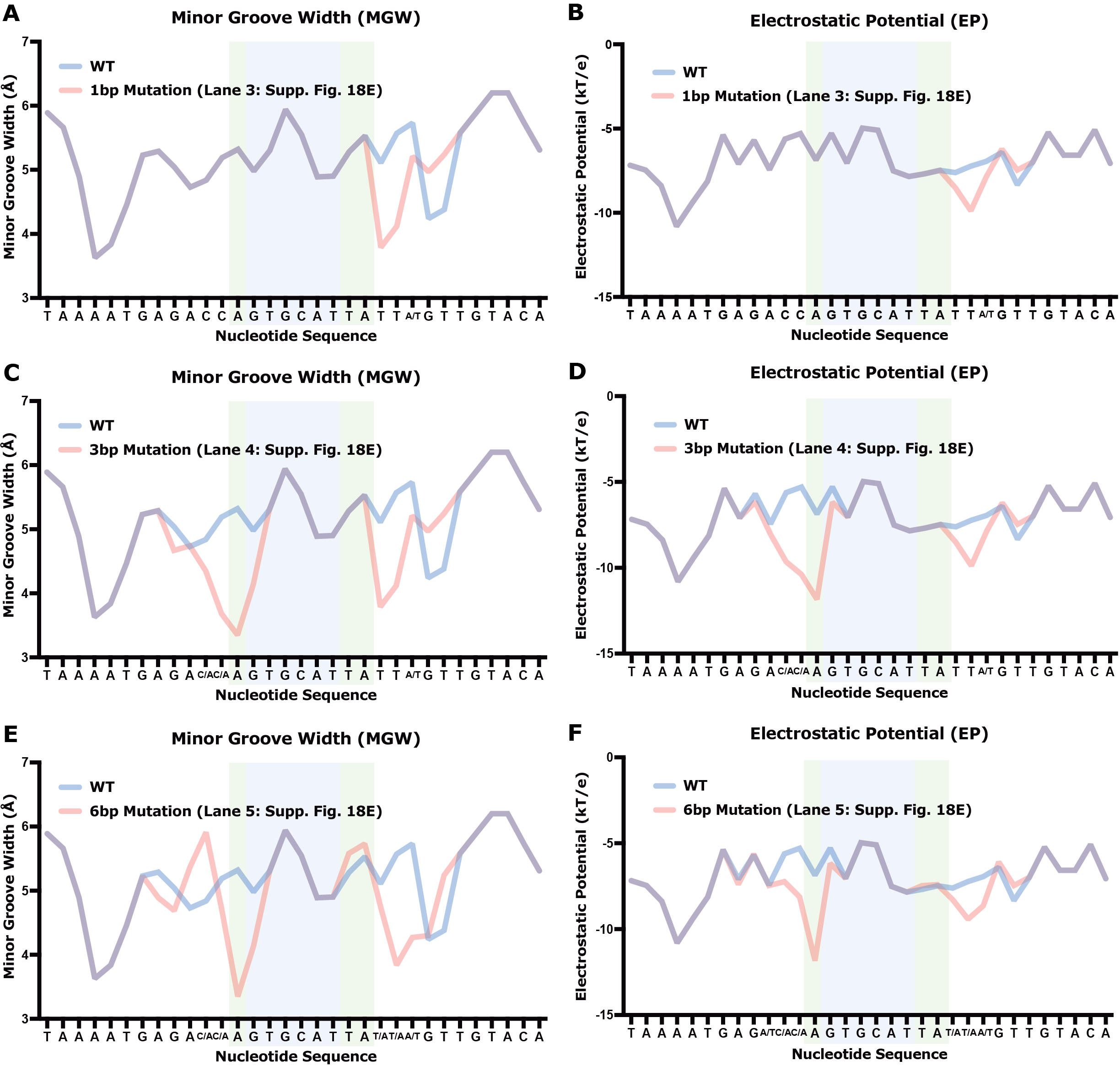


**Supplemental Figure 19: Modeled DNA shape profiles of distal shape mutations**

Calculated Minor Groove Width (MGW) and Electrostatic Potential (EP) DNA shape profiles (using DNAShapeR) of the WT (Blue) and mutated (Pink) DNA probes for electrophoretic mobility shift assays (EMSAs). Blue window highlights the GTGCAC sequence and the Green window highlights the extended motif proximal to the GTGCAC sequence on each figure. (**A-B**) MGW and EP profiles of one point mutation from **Supplemental Figure 18E**; (**C-D**) MGW and profiles of three point mutations from **Supplemental Figure 18E**; (**E-F**) MGW and EP profiles of six point mutations from **Supplemental Figure 18E**.


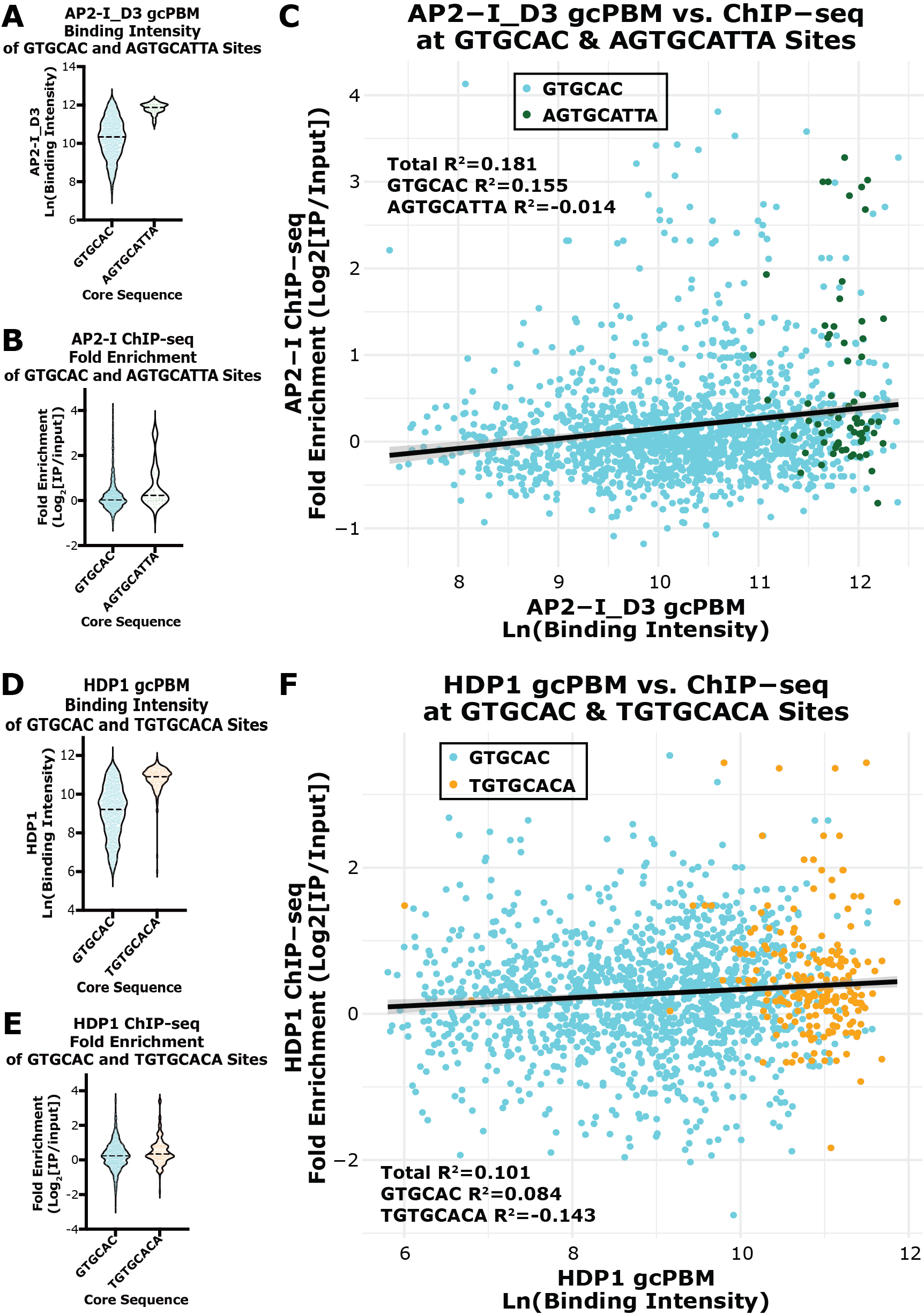


**Supplemental Figure 20: Comparing AP2-I and HDP1 binding *in vitro* and *in vivo***

**(A)** AP2-I_D3 gcPBM binding intensity across GTGCAC sites (Blue) and AGTGCATTA (Green); **(B)** AP2-I ChIP-seq fold enrichment (Log_2_[IP/Input]) across GTGCAC sites (Blue) and AGTGCATTA (Green); **(C)** Comparison of AP2-I_D3 gcPBM binding intensity and AP2-I ChIP-seq fold enrichment (Log_2_[IP/Input]) across GTGCAC sites (Blue) and AGTGCATTA (Green). *Top Right:* Pearson correlation values for all data points, only GTGCAC data points, and AGTGCATTA data points; **(D)** HDP1 gcPBM binding intensity across GTGCAC sites (Blue) and TGTGCACA (Orange); **(E)** HDP1 ChIP-seq fold enrichment (Log_2_[IP/Input]) across GTGCAC sites (Blue) and TGTGCACA (Orange); **(F)** Comparison of HDP1 gcPBM binding intensity and HDP1 ChIP-seq fold enrichment (Log_2_[IP/Input]) across GTGCAC sites (Blue) and TGTGCACA (Orange). *Top Right:* Pearson correlation values for all data points, only GTGCAC data points, and TGTGCACA data points.


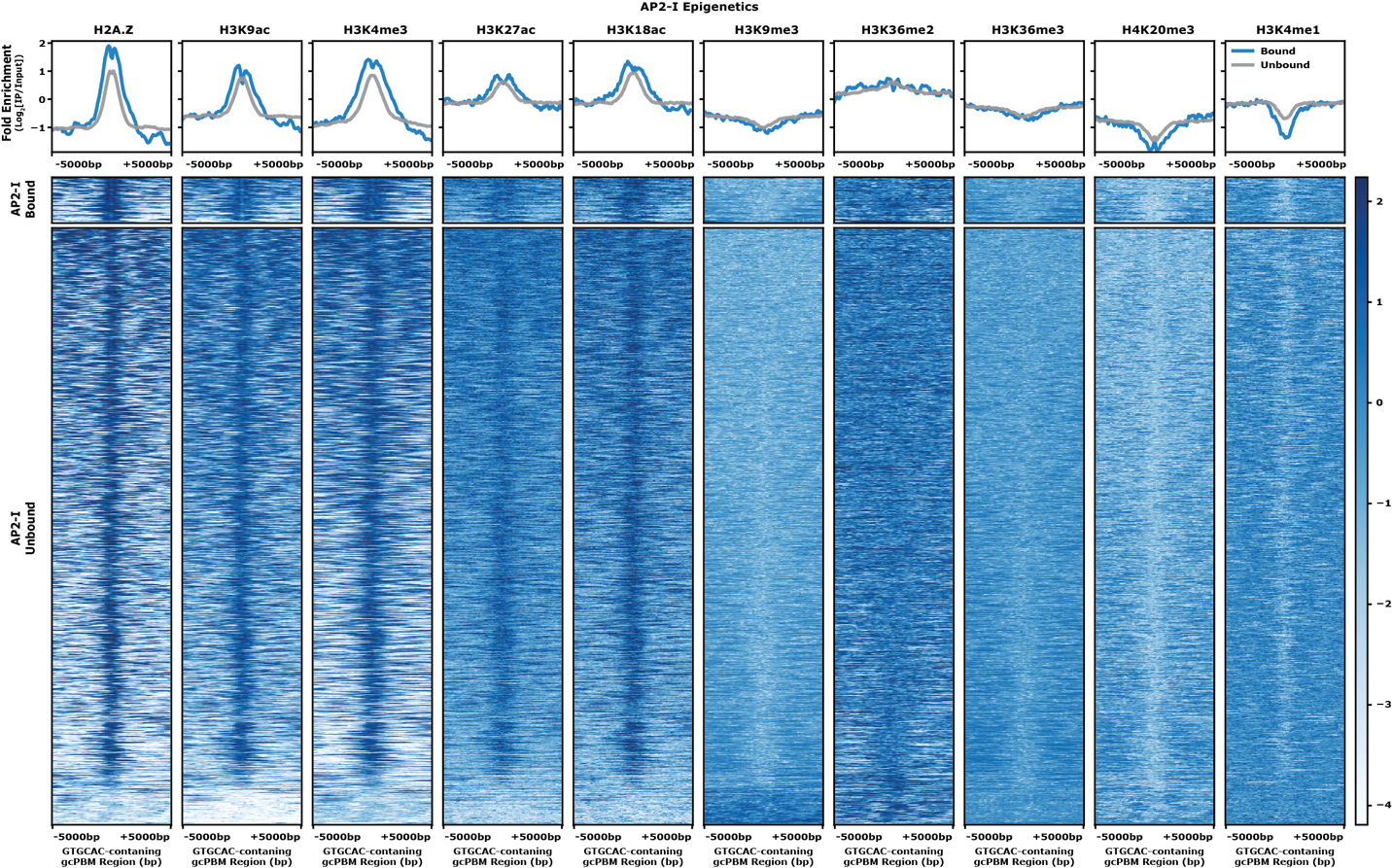


**Supplemental Figure 21: Epigenetic landscape of AP2-I ChIP-bound and ChIP-unbound GTGCAC-containing sites**

*Top:* Profile plot of the mean ChIP-seq fold enrichment (Log_2_[IP/Input]) of five activation epigenetic marks (H2A.Z, H3K9ac, H3K4me3, H3K27ac, and H3K18ac) and five repression epigenetic marks (H3K9me3, H3K36me2/3, H4K20me3, and H3K4me1) for AP2-I ChIP-bound (Blue) and ChIP-unbound (Grey) sites. *Bottom:* Heatmap of ChIP-seq fold enrichment (Log_2_[IP/Input]) of five activation epigenetic marks (H2A.Z, H3K9ac, H3K4me3, H3K27ac, and H3K18ac) and five repression epigenetic marks (H3K9me3, H3K36me2/3, H4K20me3, and H3K4me1) for AP2-I ChIP-bound (Blue) and ChIP-unbound (Grey) sites.


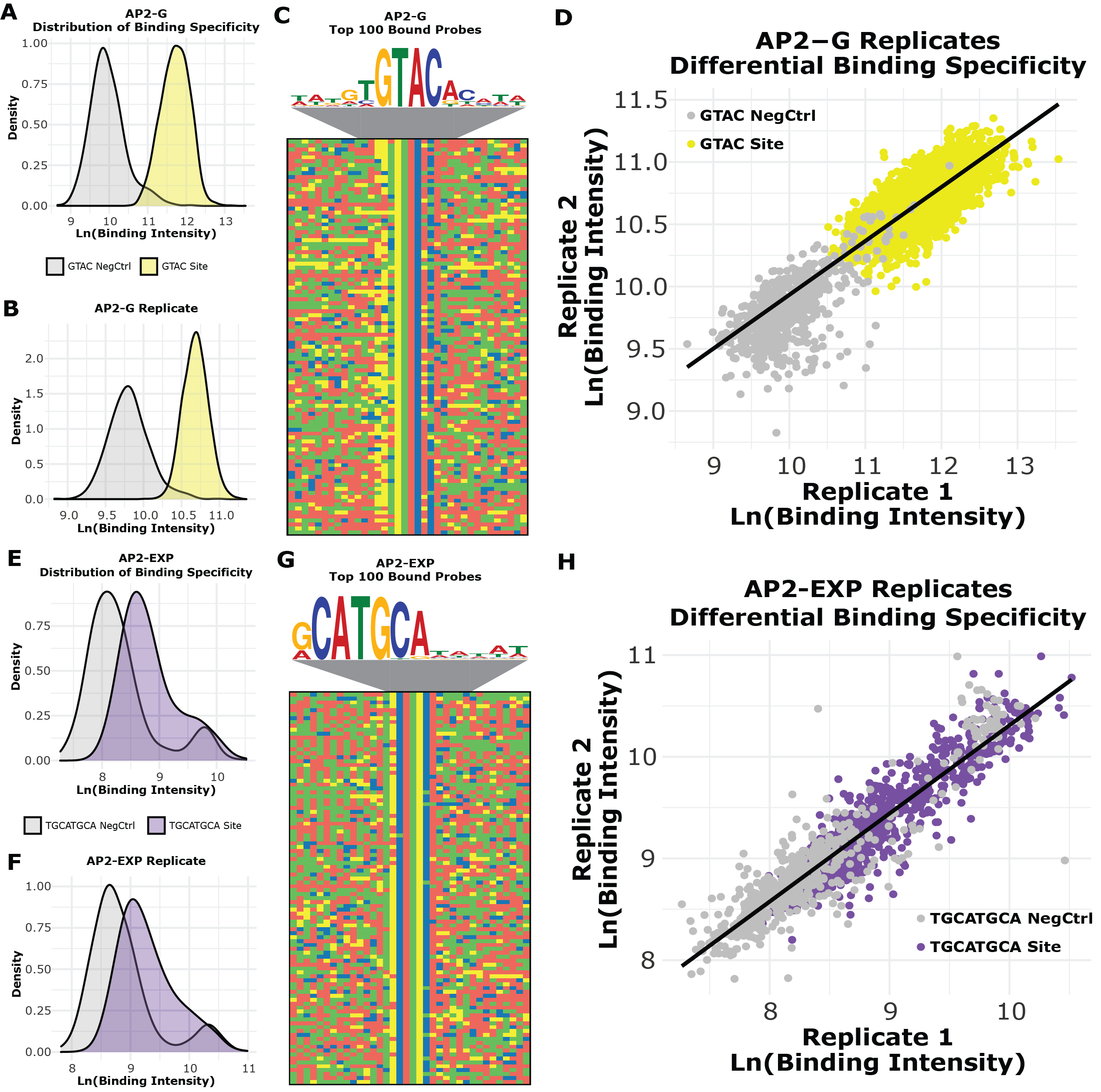


**Supplemental Figure 22: Distribution of binding specificity and replicate correlation for AP2-G and AP2-EXP**

(**A**) Density plot depicting the AP2-G binding intensities to the GTAC negative control sequences (Grey) and GTAC-containing sequences (Yellow); **(B)** Distributions of the binding intensities for AP2-G technical replicate (GTAC probes [Yellow] and negative control probes [Grey]); (**C**) The DNA motif enriched (Meme Suite: Streme) in the top 100 GTAC probes bound by AP2-G with a four-color plot of the top 100 bound GTAC probes underneath. Color representations: A (Red), C (Blue), G (Yellow), and T (Green); (**D**) Scatter plot comparing the binding intensities for AP2-G technical replicates. Each data point represents the median binding intensity across all technical replicates of the TF to a specific DNA sequence: GTAC negative control sequences (Grey) and GTAC-containing sequences (Yellow); (**E**) Density plot depicting the AP2-EXP binding intensities to the TGCATGCA negative control sequences (Grey) and TGCATGCA-containing sequences (Purple); (**F**) Density plot depicting the AP2-EXP technical replicate binding intensities to the TGCATGCA negative control sequences (Grey) and TGCATGCA-containing sequences (Purple); (**G**) The DNA motif enriched (Meme Suite: Streme) in the top 100 TGCATGCA probes bound by AP2-EXP with a four-color plot of the top 100 bound TGCATGCA probes underneath; and (**H**) Scatter plot comparing the binding intensities for AP2-EXP technical replicates. Each data point represents the median binding intensity across all technical replicates of the TF to a specific DNA sequence: TGCATGCA negative control sequences (Grey) and TGCATGCA-containing sequences (Purple).


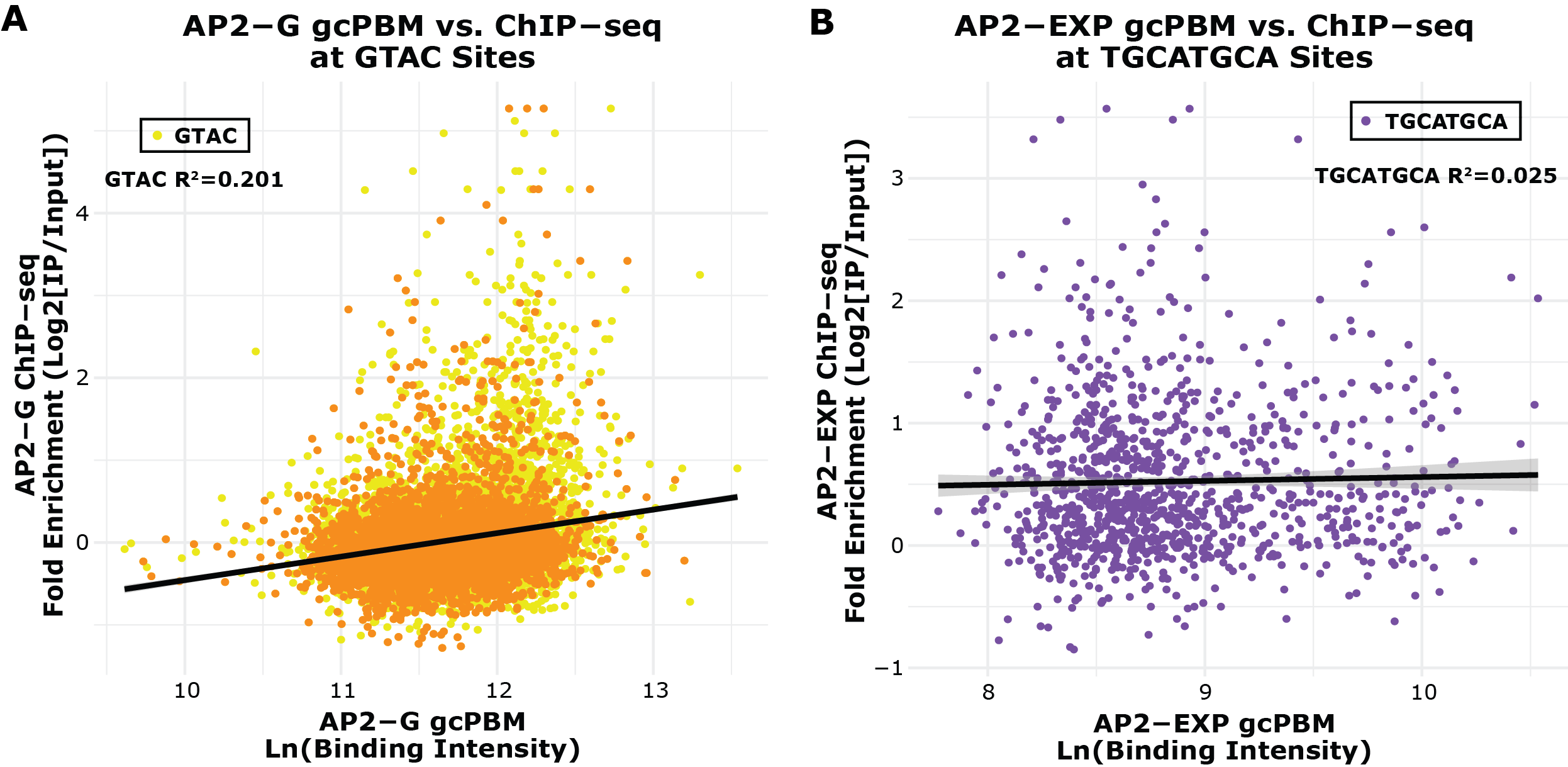


**Supplemental Figure 23: Comparing AP2-G and AP2-EXP binding *in vitro* and *in vivo***

**(A)** Comparison of AP2-G gcPBM binding intensity and AP2-G ChIP-seq fold enrichment (Log_2_[IP/Input]) across GTAC sites (Yellow). *Top Right:* Pearson correlation values for all data points; **(B)** Comparison of AP2-EXP gcPBM binding intensity and AP2-EXP ChIP-seq fold enrichment (Log_2_[IP/Input]) across TGCATGCA sites (Purple). *Top Right:* Pearson correlation values for all data points.


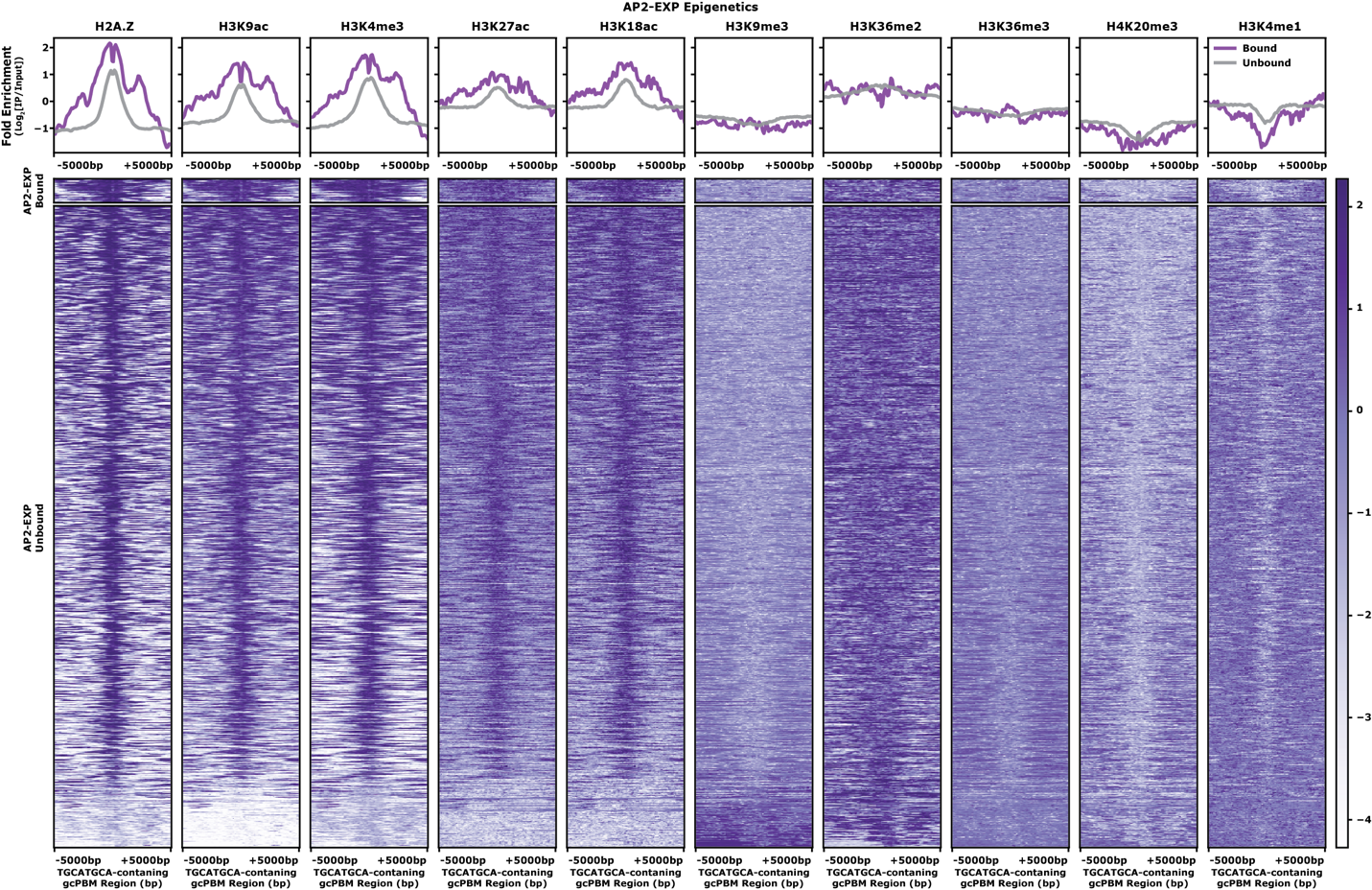


**Supplemental Figure 24: Epigenetic landscape of AP2-EXP ChIP-bound and ChIP-unbound TGCATGCA-containing sites**

*Top:* Profile plot of the mean ChIP-seq fold enrichment (Log_2_[IP/Input]) of five activation epigenetic marks (H2A.Z, H3K9ac, H3K4me3, H3K27ac, and H3K18ac) and five repression epigenetic marks (H3K9me3, H3K36me2/3, H4K20me3, and H3K4me1) for AP2-EXP ChIP-bound (Purple) and ChIP-unbound (Grey) sites. *Bottom:* Heatmap of ChIP-seq fold enrichment (Log_2_[IP/Input]) of five activation epigenetic marks (H2A.Z, H3K9ac, H3K4me3, H3K27ac, and H3K18ac) and five repression epigenetic marks (H3K9me3, H3K36me2/3, H4K20me3, and H3K4me1) for AP2-EXP ChIP-bound (Purple) and ChIP-unbound (Grey) sites.


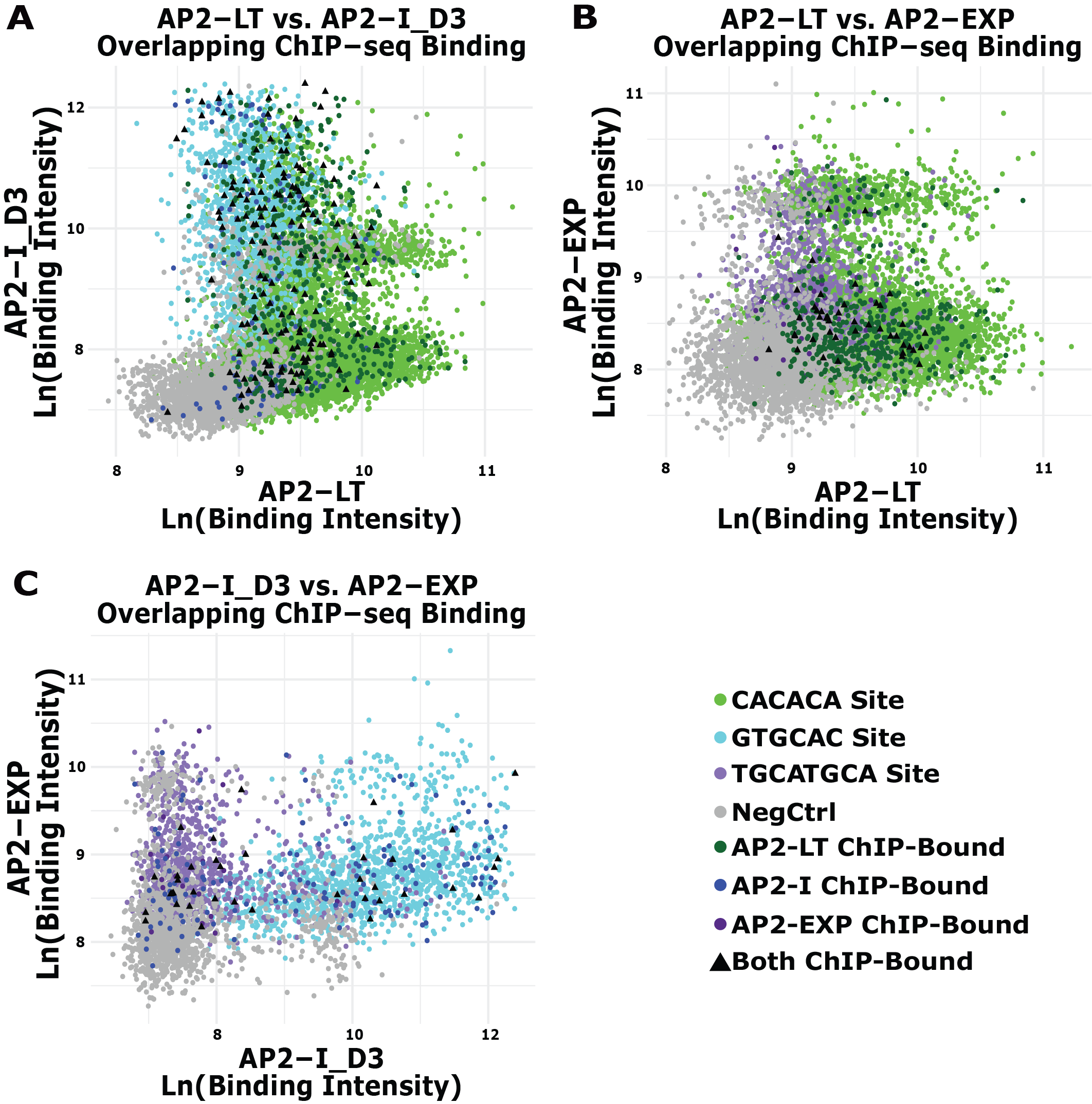


**Supplemental Figure 25: Overlapping *in vitro* binding preferences across DNA motif types**

(**A**) Comparison of the gcPBM binding intensities for AP2-LT and AP2-I_D3. Negative control sequences (Grey), CACACA-containing sequences (Green), GTGCAC-containing sequences (Blue), TGCATGCA-containing sequences (Purple), AP2-LT ChIP-bound sequences (Dark Green), AP2-I ChIP-bound sequences (Dark Blue), AP2-EXP ChIP-bound sequences (Dark Purple), and AP2-LT/AP2-I, AP2-LT/AP2-EXP, or AP2-I/AP2-EXP co-bound ChIP-bound sequences (Black triangle); (**B**) Comparison of the binding intensities for AP2-LT and AP2-EXP; and (**C**) Comparison of the binding intensities for AP2-I_D3 and AP2-EXP.

**Supplemental Files Captions:**

**Materials Reagent List:**

All reagents used in this study including company name and catalog number.

**Supplemental File 1: DNA sequence probes designed for this study**

Using position weight matrix (PWM) data from published work, all instances of each motif were identified in the *P. falciparum* genome (plasmoDB: 3D7 strain genome release v38), with a motif E-score cutoff of >0.45. Only intergenic regions (excluding telomeric regions) were used for the gcPBM design. Each TF also contained motif-containing dsDNA probes and associated non-motif-containing negative control probes represented in each gcPBM experiment. Negative control probes, randomly chosen sites without the motif of interest, for each motif type were included to observe non-specific binding of each TF to genomic regions not containing the motif of interest, which was necessary due to the A/T-rich *P. falciparum* genome. HDP1 was identified just after the design of the gcPBM experiment, therefore the HDP1 PWM was not used for the design, but was added as an additional GTGCAC-binding TF in this study. All gcPBM binding intensity data for each probe and each protein tested (with technical replicates) are included in this table.

**Supplemental File 2: DNA sequences for each DNA primer or oligo for cloning and electrophoretic mobility shift assays (EMSAs)**

Oligo sequences used for electrophoretic mobility shift assays (EMSAs). Each 36-bp sequence was taken from the gcPBM design and ordered with a 5`-biotin fused only to the forward sequence (and a non-modified reverse sequence for double stranding).

**Supplemental File 3: DNA shape distance predictions for extended motif point mutations**

The DNA oligos used to investigate the impact of DNA shape on binding were generated by the mutation design tool of TFBSshape. This tool produces oligo sequences with mutations that preserve DNA sequence or shape features according to the distance between their wild type and mutant. The sequence distance is determined by Levenshtein distance that sums the number of substitutions, deletions or insertions required to transform from a mutant to its wild type sequence. The shape distance is calculated in Euclidean distance between two normalized shape feature vectors for a wild type and its mutant sequence. The normalized shape features including Helix Twist (HelT), Minor Groove Width (MGW), Propeller Twist (ProT), and Roll are derived from DNAShapeR. Three bps on the flanks of the fixed core ‘AGTGCATTA’ were subjected to mutation, as shown in lowercase. In this case, the oligos with the maximum shape distance, with respect to the preserved sequence distance, were selected.

**Supplemental File 4: ChIP-seq MACS2 outputs for each replicate**

Three biological replicates of ChIP-seq were conducted around 40hpi (hours post invasion), which is the time of peak protein expression of AP2-LT during the 48-hour asexual blood stage. An additional ChIP-seq experiment was done with a non-modified parasite line (with the same 3D7 strain genetic background as the tagged line) to serve as a no-epitope negative control, which resulted in only one significant peak overlapping with the tagged line ChIP-seq combined replicates. This table contains the MACS2 Narrow Peaks and Fold Enrichment Table outputs for the three biological replicates and single negative control sample.

**Supplemental File 5: All core 6-mer sequences for each motif type and the number of times the 6-mer is represented on the gcPBM design**

All 6-mer sequences represented on the array design for each motif type (CACACA, GTGCAC, GTAC, and TGCATGCA). Each 6-mer DNA sequence is categorized under their motif type with the number of times it is represented in the gcPBM design (not calculating for the 6-8 replicated spots).

**Supplemental File 6: Data underlying Figure 3 transcription profile**

AP2-LT predicted putative target genes are defined by having a peak inside of the gene coding sequence or no more than 2kb upstream of the target gene transcription start site (TSS). This table includes the gene ID, gene annotation, cluster bin, and mean-centered transcript abundance (Chappell *et al.* 2020) of each gene target of AP2-LT determined by ChIP-seq.

**Supplemental File 7: Gene Ontology (GO) terms for AP2-LT gene targets for Figure 3**

This table includes the GO term IDs, GO term name, number of genes in GO term background (bgd count), number of genes from AP2-LT putative gene targets in GO term background (result count), gene IDs of all AP2-LT targets in GO term background (result gene list), percentage of AP2-LT targets over GO term background (pct of bgd), fold enrichment, odds ratio, p-value, Benjamini-adjusted p-value, and Bonferroni-adjusted p-value.
